# Age-at-Injury Determines the Extent of Long-Term Neuropathology and Microgliosis After a Diffuse Brain Injury in Male Rats

**DOI:** 10.3389/fneur.2021.722526

**Published:** 2021-09-08

**Authors:** Yasmine V. Doust, Rachel K. Rowe, P. David Adelson, Jonathan Lifshitz, Jenna M. Ziebell

**Affiliations:** ^1^Wicking Dementia Research and Education Centre, College of Health and Medicine, University of Tasmania, Hobart, TAS, Australia; ^2^Department of Integrative Physiology at University of Colorado, Boulder, CO, United States; ^3^BARROW Neurological Institute at Phoenix Children's Hospital, Phoenix, AZ, United States; ^4^Department of Child Health, University of Arizona College of Medicine – Phoenix, Phoenix, AZ, United States; ^5^Phoenix Veteran Affairs Health Care System, Phoenix, AZ, United States

**Keywords:** traumatic brain injury, TBI, concussion, aging, puberty, juvenile, age-at-injury, pathology

## Abstract

Traumatic brain injury (TBI) can occur at any age, from youth to the elderly, and its contribution to age-related neuropathology remains unknown. Few studies have investigated the relationship between age-at-injury and pathophysiology at a discrete biological age. In this study, we report the immunohistochemical analysis of naïve rat brains compared to those subjected to diffuse TBI by midline fluid percussion injury (mFPI) at post-natal day (PND) 17, PND35, 2-, 4-, or 6-months of age. All brains were collected when rats were 10-months of age (*n* = 6–7/group). Generalized linear mixed models were fitted to analyze binomial proportion and count data with R Studio. Amyloid precursor protein (APP) and neurofilament (SMI34, SMI32) neuronal pathology were counted in the corpus callosum (CC) and primary sensory barrel field (S1BF). Phosphorylated TAR DNA-binding protein 43 (pTDP-43) neuropathology was counted in the S1BF and hippocampus. There was a significantly greater extent of APP and SMI34 axonal pathology and pTDP-43 neuropathology following a TBI compared with naïves regardless of brain region or age-at-injury. However, age-at-injury did determine the extent of dendritic neurofilament (SMI32) pathology in the CC and S1BF where all brain-injured rats exhibited a greater extent of pathology compared with naïve. No significant differences were detected in the extent of astrocyte activation between brain-injured and naïve rats. Microglia counts were conducted in the S1BF, hippocampus, ventral posteromedial (VPM) nucleus, zona incerta, and posterior hypothalamic nucleus. There was a significantly greater proportion of deramified microglia, regardless of whether the TBI was recent or remote, but this only occurred in the S1BF and hippocampus. The proportion of microglia with colocalized CD68 and TREM2 in the S1BF was greater in all brain-injured rats compared with naïve, regardless of whether the TBI was recent or remote. Only rats with recent TBI exhibited a greater proportion of CD68-positive microglia compared with naive in the hippocampus and posterior hypothalamic nucleus. Whilst, only rats with a remote brain-injury displayed a greater proportion of microglia colocalized with TREM2 in the hippocampus. Thus, chronic alterations in neuronal and microglial characteristics are evident in the injured brain despite the recency of a diffuse brain injury.

## Introduction

Traumatic brain injury (TBI) is a neurological condition that commonly leads to long-term functional deficits such as impaired memory, cognition, and sensorimotor function (1). In conjunction with long-term functional deficits, TBI has also been associated with an increased risk of the development of neurodegenerative diseases such as Alzheimer's disease, chronic traumatic encephalopathy (CTE), multiple sclerosis, and Parkinson's disease (2). This chronic presentation of symptoms and increased risk of neurodegenerative disease may be a result of TBI-induced cascades that manifest as enduring neuronal injury and inflammation, as observed both clinically and experimentally (3–5). Worldwide, the rate of TBI is high, wherein a recent meta-analysis indicated the highest incidence to be in Australasia, at an estimated 415 in every 100,000 people (6). Across the lifespan, TBI is most prevalent in early-life due to domestic violence/child abuse and falls (7–10), with secondary peaks in adolescence and aged individuals attributed to increased risk-taking behaviors (i.e., driving) and falls, respectively (7). Due to the high prevalence of 18- to 25-year-old male individuals that sustain a TBI (7), clinical and experimental research has focused on the behavioral and neuropathological outcomes in young adult males. There is cumulative research in humans and various animal models that demonstrate a TBI at any age can result in long-term sensorimotor, cognitive, and endocrine symptoms in addition to neuropathology and glial activation, but it remains unclear whether age-at-injury affects these outcomes (11–16).

An explanation for the extent of TBI neuropathology with respect to age-at-injury may involve glial changes (17). Glia exhibit an altered function with aging and, thus, may influence the TBI neuropathology to a different extent based on the stage of development when the injury is delivered. Microglia and astrocytes become dysfunctional with age, which results in an increased pro-inflammatory profile as well as loss of homeostatic functions (18–21). This dysfunctional aged phenotype of glia may promote the vulnerability of neuronal injury as well as modify the magnitude and duration of the inflammatory response (22).

Competing theories exist for when the brain is most vulnerable to TBI, as the immature brain is more neuroplastic than the adult and aged brain. Meaning the immature brain may have greater capacity for self-repair after an injury and, hence, minimal functional impairment (23). On the contrary, underdevelopment of the young brain may increase vulnerability to long-term deficits after a brain injury, because of a potential interruption of critical developmental processes (24, 25). With regard to the aging brain, tissue properties (e.g., stiffness, atrophy) may impart susceptibility to damage after a TBI, which can worsen functional outcomes in comparison to injury in juveniles and adults (26, 27). More research is required to better understand the relationship between age and enduring pathology from a TBI which might inform possible therapeutic intervention.

In our prior work in rats, age-at-injury was associated with persistent motor deficits on the beam walk, anxiety-like behavior in the open field, and spatial memory deficits with novel objects (28). Here, we investigated whether neuropathology, in those same animals, was related to the recency (2-, 4-, 6-months of age-at-injury) or remoteness (17- or 35-days old at injury; PND17, PND35) of a TBI, where neuropathology was inclusive of neuronal, astroglial, and microglial changes. We hypothesized that neuropathology and gliosis is evident after a diffuse TBI and the extent of pathology is determined by age-at-injury.

## Methods

### Experimental Design, Injury Induction, and Tissue Collection

Male Sprague-Dawley rats (Harlan Laboratories, Inc., Indianapolis, Ind., USA) were received as a single shipment at post-natal day (PND) 10, with the dam, and acclimated for 7-days prior to experimentation. This report is part of a larger study where rats (*n* = 81) were assigned to age-at-injury groups upon arrival. Rats underwent behavioral assessments following TBI with results being previously published (28). A midline fluid percussion injury (mFPI) was used to experimentally produce a diffuse TBI at juvenile (PND17), adolescent (PND35), young adult (2-months-old), or mature adult (4- and 6-months-old) ages compared to naïve rats. Group allocation, injury induction, and housing have been previously described (28). Briefly, rats were anesthetized with isoflurane (5% mixed with 100% O_2_ at 1 L/min) for 5 min and the head was secured in a stereotaxic frame via the ear and bite bars. Anesthesia and body temperature were maintained throughout the surgery by a nosecone (2.5% isoflurane mixed with 100% O_2_ at 0.4 L/min) and a Deltaphase isothermal heating pad, respectively. A midline incision was made from between the eyes to just behind the ears with the fascia scraped from the skull. Various sizes of trephines were used based on the ratio of the size of the craniotomy to the size of the skull [3.0 mm (PND 17), 4.0 mm (PND35), 4.8 mm (2-, 4-, 6-months) (29)] to remove a circular piece of the skull on the sagittal suture, half-way between bregma and lambda, with the dura remaining intact. The female Luer-Loc injury hub was cut from a needle and secured over the craniotomy site using cyanoacrylate gel followed by methyl-methacrylate. The posterior and anterior edges of the incision were closed and topical Lidocaine ointment applied to the area. Rats were placed in a recovery cage warmed upon a heating pad and monitored until ambulatory.

After 60–90 min of recovery, rats were re-anesthetized with isoflurane (5% mixed with 100% O_2_ at 1 L/min) for 5 min in preparation for injury induction. The hub and dura were visually inspected for obstructions or blood and then filled with saline. The female Luer-Loc on the rats was connected to the male Luer-Loc on the fluid percussion injury device; PND17 rats were connected using a Luer-Loc extension tube. Traumatic brain injury was induced by releasing the pendulum onto the fluid-filled cylinder, producing a pressure pulse (atmospheres) depending on age and body-weight: 1.5 atm (PND17), 1.9 atm (PND35), 2.1 atm (2-months), 2.2 atm (4-months), 2.7 atm (6-months). The time, post-injury, before the rats regained the righting reflex, as well as the duration of apnea and seizures, were recorded. The righting reflex times in this study ranged between 5 and 10 min, which represents an injury with known acute motor deficits. Once righted, the rats were re-anesthetized with isoflurane (5% mixed with 100% O_2_ at 1 L/min) for 5 min and the injury hub was removed. Before closing the incision, the wound was cleaned with saline and the craniotomy site and underlying dura were inspected for hematoma, herniation, and dural breach.

Rats recovered in warmed cages until ambulatory (approx. 5 and 15 min) before being returned to the dam (PND17) or home cage and the cage placed back in the colony. Post-surgical evaluations were conducted on the rats daily for 3 days via physical examination of behavior, appearance, suture- or staple-site, and weight as well as for any signs of pain or distress. The weight of all rats was recorded weekly for 10-months; no statistical differences in weight were noted (28). Appropriate measures were taken to minimize animal pain and distress associated with surgery and brain injury. All animal care and experimental procedures were approved by the Institutional Animal Care and Use Committees (IACUC) at the University of Arizona (Phoenix, AZ, USA) and conducted in accordance with the Association for Assessment and Accreditation of Laboratory Animal Care International and NIH guidelines. This research is reported according to the ARRIVE (Animal Research: Reporting *in vivo* Experiments) guidelines. Twelve rats were excluded from the study entirely: (1) one rat developed an epithelial tumor; (2) one rat aspirated after the forced swim behavioral task; (3) one rat was incorrectly sexed during initial screening upon arrival and was, thus, removed along with its cage mate;(4) two 6 month rats died after injury; (5) five rats were excluded as surgical failures.

Each rat was housed with another rat assigned to a different injury group, such that cage mates did not receive an injury at the same time. Injury groups were assigned prior to experimentation in order to achieve randomization. Power calculations were conducted to determine sample size for adequate detection of injury-induced pathology whilst minimizing animal numbers. A battery of behavior tests was conducted on these animals at 1.5-, 3-, 5-, 7-, and 10-months of age to assess vestibulomotor function, spatial memory, anxiety, and depressive-like behavior, as previously described [(28); Supplementary Figure 1]. Tissue was collected at 10-months of age where rats were transcardially perfused with 10% formalin (Millipore Sigma, USA). The brain remained *in situ* for approximately 1 h before being dissected from the cranium and drop-fixed in 10% formalin overnight, then transferred into 0.02% sodium azide (NaN_3_) in phosphate buffered saline (PBS). Brains (*n* = 36; naïve, *n* = 5; PND17, *n* = 6; PND35, *n* = 7; 2 months, *n* = 6; 4 months, *n* = 6; 6 months, *n* = 6) were shipped to the University of Tasmania (Hobart, Tasmania, Australia) for sectioning and tissue analysis (Supplementary Figure 1). Brains were sectioned in the coronal plane at 40 μm on a vibratome and tissue was collected in a 24-well plate and stored at 4°C in PBS with 0.02% sodium azide prior to performing immunohistochemistry. During the immunohistochemical analyses, the investigator was blinded to the age-at-injury group of the sample.

### Immunohistochemical Staining for Neuropathology and Glial Activation

#### Nickel-Enhanced 3,3-Diaminobenzidine Immunohistochemistry of Neuropathological Profiles and Astrogliosis

Three sections per brain were stained with APP, SMI32, SMI34, and Phosphorylated TAR DNA-binding protein 43 (pTDP-43) for neuronal pathology (approximate location anterior/posterior to Bregma: −0.37, −2.26, −4.38 mm) and Glial fibrillary acidic protein (GFAP) immunoreactivity (approximate location anterior/posterior to Bregma: −3.68, −4.03, −4.38 mm). Tissue sections were washed in PBS with 0.01% tween-20 (PBST) then placed in a blocking solution for 90 min (see Table 1). Sections were incubated with the primary antibody diluted in 1% block solution overnight at 4°C (see Table 1). Sections were washed in PBST and incubated in the corresponding biotinylated secondary antibody diluted in blocking solution for 1 h (see Table 2). After washing, sections were placed in 3% hydrogen peroxide for 10 min. Sections were rinsed and placed in Avidin-Biotin Complex (ABC, Vector) for 30 min. Tissue was washed a final time in PBST and placed in nickel-enhanced 3,3-diaminobenzidine (DAB) (Vector, cat no. SK4100) for 5 min then transferred to a plate of tap water. Sections were mounted on glass slides (Dako, Denmark) and left to air-dry (~30 min) before being dehydrated in graded ethanol, cleared in xylene, and cover slipped with DePeX. For each staining run, a negative control (no primary antibody) was included. For each stain all sections from each animal were stained in one batch to prevent variation.

**Table 1:** Antibody and blocking solution information used when conducting nickel-enhanced 3,3-diaminobenzidine (DAB) and fluorescent-labeling immunohistochemistry.

**Primary antibody**	**Company**	**Concentration**	**Wash solution**	**Blocking solution**
Rabbit anti-APP C-terminus	Invitrogen, cat no. RB-9023-P0	1:1,000	PBST	10% NHS + 1.5% TX-100 in PBS
Mouse anti-SMI34	Biolegend, cat no. 835502	1:2,000	PBST	4% NHS + 0.25% TX-100 in PBS
Mouse anti-SMI32	Biolegend, cat no. 801701	1:2,000	PBST	4% NHS + 0.25% TX-100 in PBS
Rabbit anti-pTDP-43 s409/410	Cosmo, cat no. TIP-PTD-P02	1:2,000	PBST	4% NHS + 0.25% TX-100 in PBS
Rabbit anti-GFAP	Dako, cat no. Z0334	1:5,000	PBS	4% NHS in PBS
Rabbit anti-Iba1	Wako, cat no. 019-19741	1:1,000	PBS	4% NGS in PBS
Rat anti-CD68	BioRad, cat no. MCA1957GA	1:500	PBS	4% NGS in PBS
Sheep anti-TREM2	R and D Systems, cat no. AF1729	1:500	PBS	4% NGS in PBS

*PBST, PBS + 0.01% tween-20; TX-100, TritonX-100; NHS, normal horse serum; NGS, normal goat serum*.

**Table 2:** Secondary antibody information used when conducting nickel-enhanced 3,3-diaminobenzidine (DAB) and fluorescent-labeling immunohistochemistry.

**Secondary antibody**	**Company**	**Concentration**
Horse anti-rabbit biotinylated	Vector, cat no. BA-1100	1:1,000
Horse anti-mouse biotinylated	Vector, cat no. BA-2000	1:1,000
Donkey anti-rabbit Alexa Fluor 594	Invitrogen, cat no. A-21207	1:1,000
Donkey anti-rat Alexa Fluor 488	Invitrogen, cat no. A21208	1:1,000
Donkey anti-sheep Alexa Fluor 488	Invitrogen, cat no. A-11015	1:1,000

Sections incubated with rabbit anti-TDP-43 phosphorylated s409/410 (pTDP-43) required citric acid buffer (pH 4.0) antigen retrieval for 20 min at 80°C and left to cool to room temperature (RT) prior to being placed in blocking solution. These sections were counterstained with nuclear fast red (70% ethanol for 5 min, nuclear fast red for 10 min and tap water for 2 min) prior to dehydration with ethanol. Slides were then cleared in xylene and cover slipped with DePex mounting medium (Dako, Denmark) using the Dako automated cover slipper (Dako, Denmark).

#### Double Labeling of Microglia and Phenotypic Markers

Two sections per brain (approximate location anterior/posterior to Bregma: −2.26, −4.38 mm) were stained for microglia and colocalization with functional markers. Tissue sections, adjacent to those stained with DAB, were washed in PBS and placed in a blocking solution of 4% normal goat serum (NGS) in PBS for 90 min. Sections were then incubated in the primary antibody diluted in 1% block solution overnight at 4°C (see Table 1). After being washed in PBS, the tissue was incubated in secondary antibodies for 1 h (see Table 2). Followed by a final wash in PBS, the sections were then mounted on slides and cover slipped with fluoromount fluorescent mounting medium (Dako, Denmark). Slides were stored at 4°C shielded from light until fluorescent confocal microscopy. Negative controls were included in each run to ensure there was no cross-reactivity of antibodies.

### Image Acquisition and Data Analyses

#### Regions of Interest Selected for Analyses of Neuropathological Profiles and Glial Response

The white matter of the corpus callosum (CC) is known to harbor neuronal pathology after a TBI as the angle of axons entering the CC make them particularly vulnerable to injury (30). Neuronal injury is also reported in the primary sensory barrel field (S1BF), which is associated with sensory deficits that are commonly described after a TBI (31–33). The cingulate cortex connects to the hippocampus, which, together, form the majority of the memory circuitry (34). The periventricular white matter that surrounds the lateral ventricle is associated with the sensory circuitry (35). The dorsolateral entorhinal cortex is also involved with the sensory and memory circuitry (36, 37). The zona incerta is a subthalamic nucleus that connects many brain regions, including the sensory- and memory-related structures which are regularly reported to exhibit functional impairments and neuropathology after a TBI (38–40). The ventral posteromedial nucleus (VPM) is part of the thalamus, that, in conjunction with the zona incerta, connects with many brain regions, such as the S1BF and hippocampus (41, 42). The posterior hypothalamic nucleus is also involved with the sensory and memory circuitry, including specific glial roles in regulating metabolism (43), which is known to be affected post-injury as a result of endocrine dysfunction (43–45).

Bregma levels (approximate location anterior/posterior to Bregma: −0.37, −2.26, −4.38 mm) stained for neuronal pathology contained the CC, S1BF, and hippocampus. Bregma levels (approximate location anterior/posterior to Bregma: −3.68, −4.03, −4.38 mm) stained for GFAP contained the S1BF, hippocampus, cingulate cortex, periventricular white matter, dorsolateral entorhinal cortex, and zona incerta. Finally, bregma levels examined for microglial activation (approximate location anterior/posterior to Bregma: −2.26, −4.38) contained the S1BF, hippocampus, VPM, and posterior hypothalamic nucleus. The only regions investigated for amyloid precursor protein (APP) and neurofilament neuronal pathology (SMI34, SMI32) were the CC and S1BF. As pTDP-43 neuronal proteopathy occurs in the perikarya and proximal axon, pTDP-43 was examined in cell bodies of the S1BF and hippocampus. Astrocyte and microglial activation were also investigated in regions of neuronal pathology (S1BF, hippocampus). When neurons become damaged, communication between brain regions can be compromised, without overt neuronal pathology, but evident in glial changes (46). As such, the connecting regions examined for astrocyte activation were the cingulate cortex, periventricular white matter, dorsolateral entorhinal cortex, and zona incerta. For microglia activation, the connecting structures were the zona incerta, VPM, and posterior hypothalamic nucleus. Neuronal pathology was not quantified in the cingulate cortex, periventricular white matter, dorsolateral entorhinal cortex, zona incerta, and VPM as there was no overt pathology in these regions (Supplementary Figure 2).

#### Brightfield and Confocal Microscopy of Coronal Tissue Sections Stained for Neuropathology and Glia

Photomicrographs of DAB-stained tissue were taken using the ZEISS Axio Lab.A1 with an attached 6-megapixel ZEISS Axiocam 506 color camera using the 20x/0.4 objectives (ZEISS, Germany). For fluorescent-stained tissue, z-stack images (1 image taken per 1 μm of optical thickness) were captured via the Volocity 6.3 software using the Perkin-Elmer Ultra*VIEW* VoX system, involving an inverted Ti Eclipse microscope (Nikon, Japan) with a CSU-X1 spinning disk confocal scanner (Yokogawa Electric Corporation, Japan), plan apochromatic 20x/0.75 objective (Nikon, Japan), and excitation lasers/emission filters of wavelength 488/525 and 561/615.

#### Segmentation of GFAP-Labeled Astrocytes

One image was taken per region of interest (ROI) per hemisphere from three sections per animal to yield six images per animal per ROI which were analyzed for GFAP-labeled astrocytes. Images were converted from TIFFs to 8-bit TIFFs for segmentation, using the Fiji plugin, imageSURF (47). Segmentation distinguishes the positive staining from the background that were quantified to yield the number of GFAP-positive pixels per image. The number of GFAP-positive (GFAP+) pixels in each image is expressed as the number GFAP+ pixels/mm^2^ which is averaged across each group with 95% confidence intervals (CI).

#### Manual Counts of Neuropathological Profiles and Microglial Phenotypes

For counts of APP, SMI32, SMI34, and pTDP-43 neuronal pathology, one image was taken per ROI per hemisphere from three sections per animal to yield six images per animal per ROI. The number of neuropathological profiles were counted in each image and expressed as the number of neuropathological profiles/mm^2^ which is averaged across each group with 95% CI.

In order to count microglial morphologies and colocalization with functional markers, one image was taken per hemisphere from two sections per animal to yield four images per animal per ROI. A 1 mm^2^ grid was placed across the image and all microglia within every other square counted, with a target of 100 microglia, using the cell counter tool; similar to our previously published work (48). The total number of microglia and the number of deramified microglia were summed for all four images per animal. The number of deramified microglia was then divided by the total number of microglia cells to yield a proportion of deramified microglia out of the total number of cells counted in all four images. The proportion of deramified microglia per animal was then averaged across each group with 95% CI. The proportion of microglia colocalized with functional markers (CD68, TREM2) was calculated by counting the total number of microglia and the number of microglia colocalized with either CD68 or TREM2. The number of colocalized microglia was then divided by the total number of microglia to yield a proportion of colocalized microglia out of the total number of cells counted. The proportion of colocalized microglia per animal was then averaged across each group with 95% CI.

### Statistical Analysis

The binomial proportion of total cells (microglia) that were deramified or colocalized with CD68 or TREM2 was estimated using logistic regression in a generalized linear mixed effects model. Cell counts were made within the S1BF, hippocampus, VPM nucleus, zona incerta, and posterior hypothalamic nucleus, so a random intercept was fitted for each animal to account for this clustering in the data. We used the “lme4” package (49) in the R statistical (50) computing environment.

Counts of APP, SMI34, and SMI32 neuropathology were conducted in the CC and S1BF. Counts of pTDP-43 pathology were performed in the S1BF and hippocampus. Counts of GFAP-positive pixels were conducted in the S1BF, hippocampus, cingulate cortex, periventricular white matter, dorsolateral entorhinal cortex, and zona incerta. For count data we fitted generalized linear mixed effects models with random intercepts to account for clustering in the data, but used the “glmmTMB” R package (51) so that we could assess different error distributions. We fitted Poisson and negative binomial models (linear and quadratic parameterizations) and selected the best model using Aikake's information criterion (52). Consequently, we fitted negative binomial models (quadratic parameterization) for APP, SMI34, SMI32, and pTDP-43 counts; whilst we fitted Poisson models (linear parameterization) for counts of GFAP+ pixels. For numerical stability of pTDP-43 counts we added 1 to all counts and used a log link function, since the naïve controls expressed no TDP43. An alternative was to fit a Gaussian model, but this mode underestimated standard errors and gave poor coverage of CI.

Likelihood ratio tests were used to compute *p*-values. Estimated marginal means and *post-hoc* contrasts were computed with the “emmeans” R package (53), with corrections for multiple comparisons to appropriately control Type 1 errors using Dunnett's method.

## Results

This study investigated whether age-at-injury influenced neuropathology and glial activation at 10-months of age in male rats. For this, 36 male rats were either subjected to a single diffuse TBI at PND17, PND35, 2-, 4-, or 6-months of age or allowed to age in the naïve state. At 10-months of age, all rats were prepared for brain immunohistochemical studies reported here. Neurobehavioral outcomes in these rats have been reported elsewhere (28).

### The Extent of APP Axonal Pathology Was Greater in Brain-Injured Groups Compared With Naïve in the CC and S1BF, Regardless of Whether the TBI Was Recent or Remote

**Figure 1:**
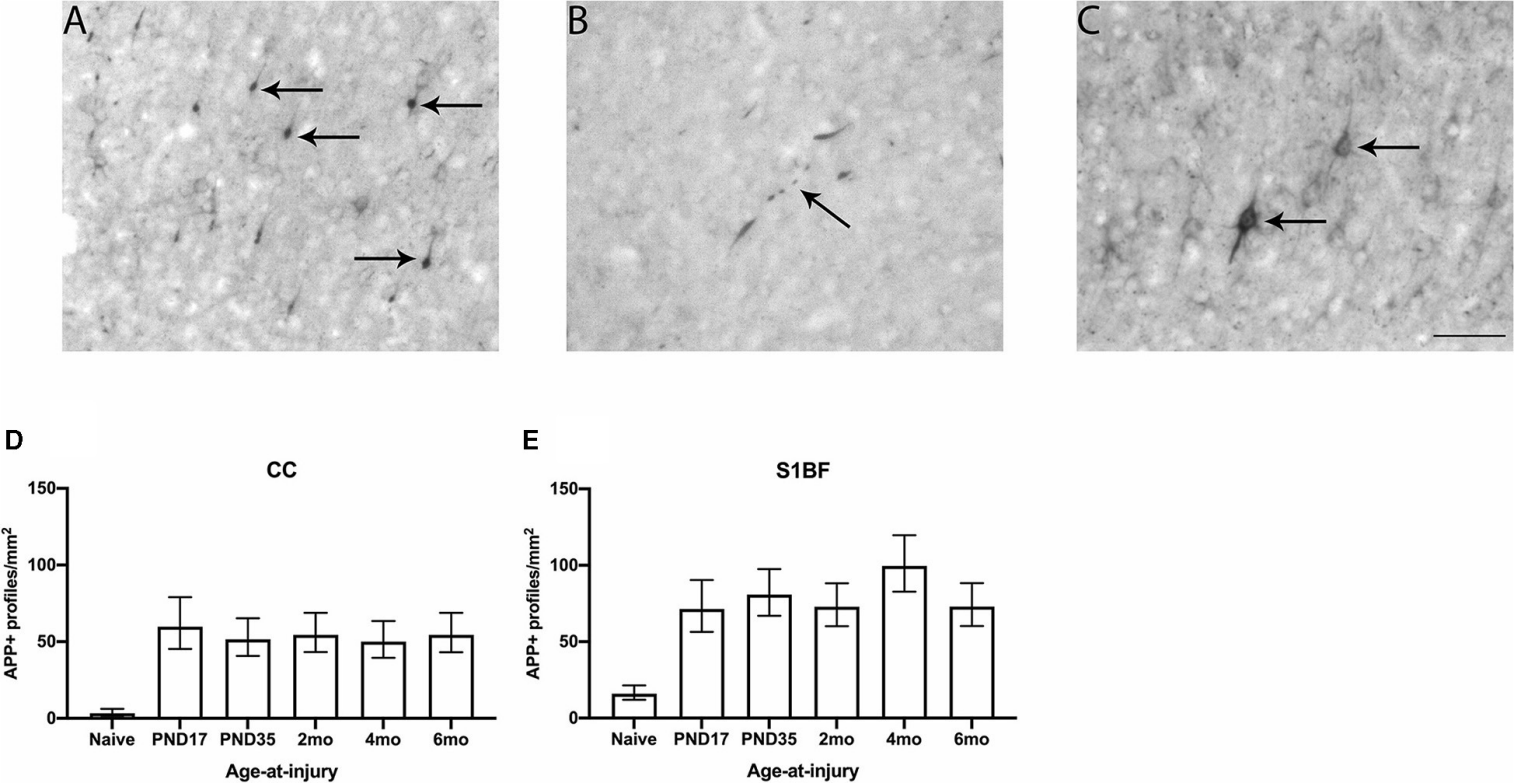
APP neuronal profiles were quantified in the CC and S1BF. Indicative images of neuronal pathology visualized by APP immunohistochemistry were taken in the primary sensory barrel field (S1BF) of rats brain-injured at 6-months of age. Quantitative analysis included bulbs **(A)**, blebbing **(B)**, and cell bodies **(C)** indicated by the black arrows (scale bar = 50 μm). Counts of neuronal pathology are expressed as the number of APP-positive (APP+) profiles/mm^2^ (mean ± 95%CI) in the corpus callosum (CC) and S1BF. Significantly more APP+ profiles were observed following mFPI, regardless of age-at-injury, compared to naïves in both the CC **(D)** and the S1BF **(E)**.

A hallmark of TBI is the accumulation of APP within neuronal axons and somas (5, 54). Visualization of this axonal transport disruption in the CC and S1BF was achieved via APP immunohistochemistry. Pathological profiles were defined as bulbs, blebbing, or cell bodies which are depicted in the S1BF (Figures 1A–C). The number of APP-positive (APP+) profiles were quantified in each image taken of the CC and S1BF per mm^2^ (Figures 1D,E). Animals in the injured groups had significantly more APP+ profiles than naïve controls ($\chi_{5}^{2}$ = 70.7; *p* < 0.001), but APP+ profiles in age-at-injury groups varied significantly by region ($\chi_{5}^{2}$ = 29.8; *p* < 0.001) (see Supplementary Table 1 for estimated counts and their 95% CIs; illustrated in Figures 1D,E). These quantitative data demonstrate that neuropathology is evident after a single TBI, regardless of whether the TBI was recent or remote.

### The Extent of SMI34 Neurofilament Pathology Was Greater in Brain-Injured Groups Compared With Naïve in the CC and S1BF, Regardless of Whether the TBI Was Recent or Remote

**Figure 2:**
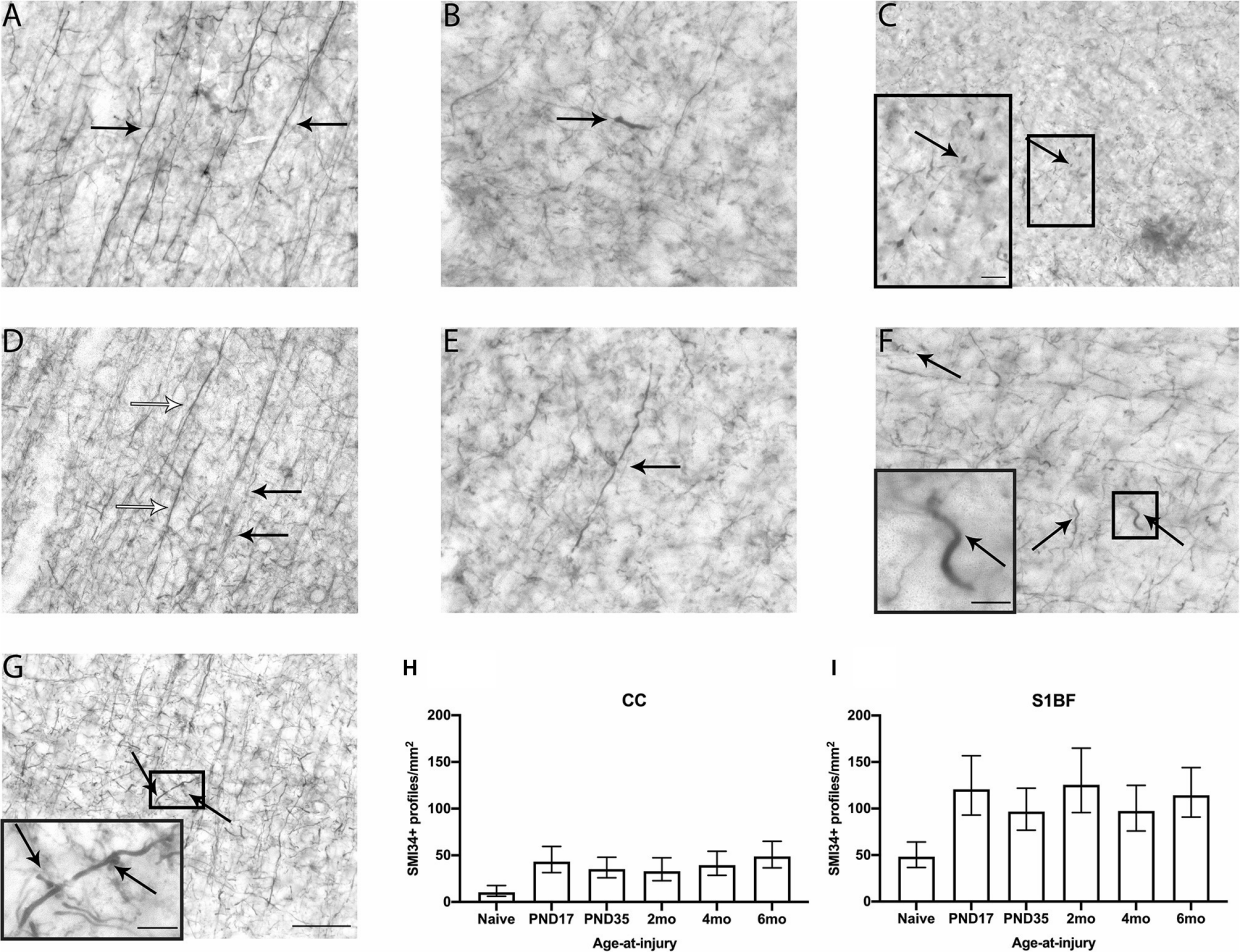
SMI34 axonal profiles were quantified in the CC and S1BF. Indicative images of healthy axons **(A)** and axonal pathology visualized by SMI34 immunohistochemistry were taken in the primary sensory barrel field (S1BF) of rats brain-injured at 6-months of age. Analysis quantified bulbs **(B)**, blebbing **(C)**, thickened axons (white arrows; **D**) or undulated axons (black arrows; **D**), thickened and undulated axons **(E)**, corkscrews **(F)**, and rings **(G)** indicated by the arrows (scale bar = 50 μm, inset = 10 μm). Counts of axonal pathology are expressed as the number of SMI34+ profiles/mm^2^ (mean ± 95%CI) in the corpus callosum (CC) and S1BF. There were significantly more SMI34-positive (SMI34+) profiles observed post-injury, regardless of age-at-injury, compared to naïves in both the CC **(H)** and the S1BF **(I)**.

Axonal pathology was assessed via changes to the axonal cytoskeleton, particularly the neurofilament network which provides the axon with caliber and integrity (55, 56). Immunohistochemistry against phosphorylated neurofilament heavy chain (SMI34) was used to visualize cytoskeletal axonal changes in the CC and S1BF. SMI34 staining in healthy axons of naïve brain appears as thin and straight fibers in the S1BF (Figure 2A), and in response to brain injury, the cytoskeleton and axon exhibit bulbs, blebbing, thickening, undulations, corkscrews, and rings (Figures 2B–G). Cytoskeletal changes were quantified in each image taken as the number of SMI34-positive (SMI34+) profiles per mm^2^ in both the CC and S1BF (Figures 2H,I). Animals in the injured groups had significantly more SMI34+ profiles than naïve controls ($\chi_{5}^{2}$ = 24.0; *p* < 0.001), but SMI34+ profiles in age-at-injury groups varied significantly by region ($\chi_{5}^{2}$ = 16.4; *p* < 0.01) (see Supplementary Table 2 for estimated counts and 95% CIs; also illustrated in Figures 2H,I). These quantitative data demonstrate that neurofilament axonal pathology is present after a TBI, regardless of age-at-injury.

### The Extent of SMI32 Neurofilament Pathology Was Greater in Brain-Injured Groups Compared With Naïve in the CC, Regardless of Whether the TBI Was Recent or Remote, but Was Determined by Age-at-Injury in the S1BF

**Figure 3:**
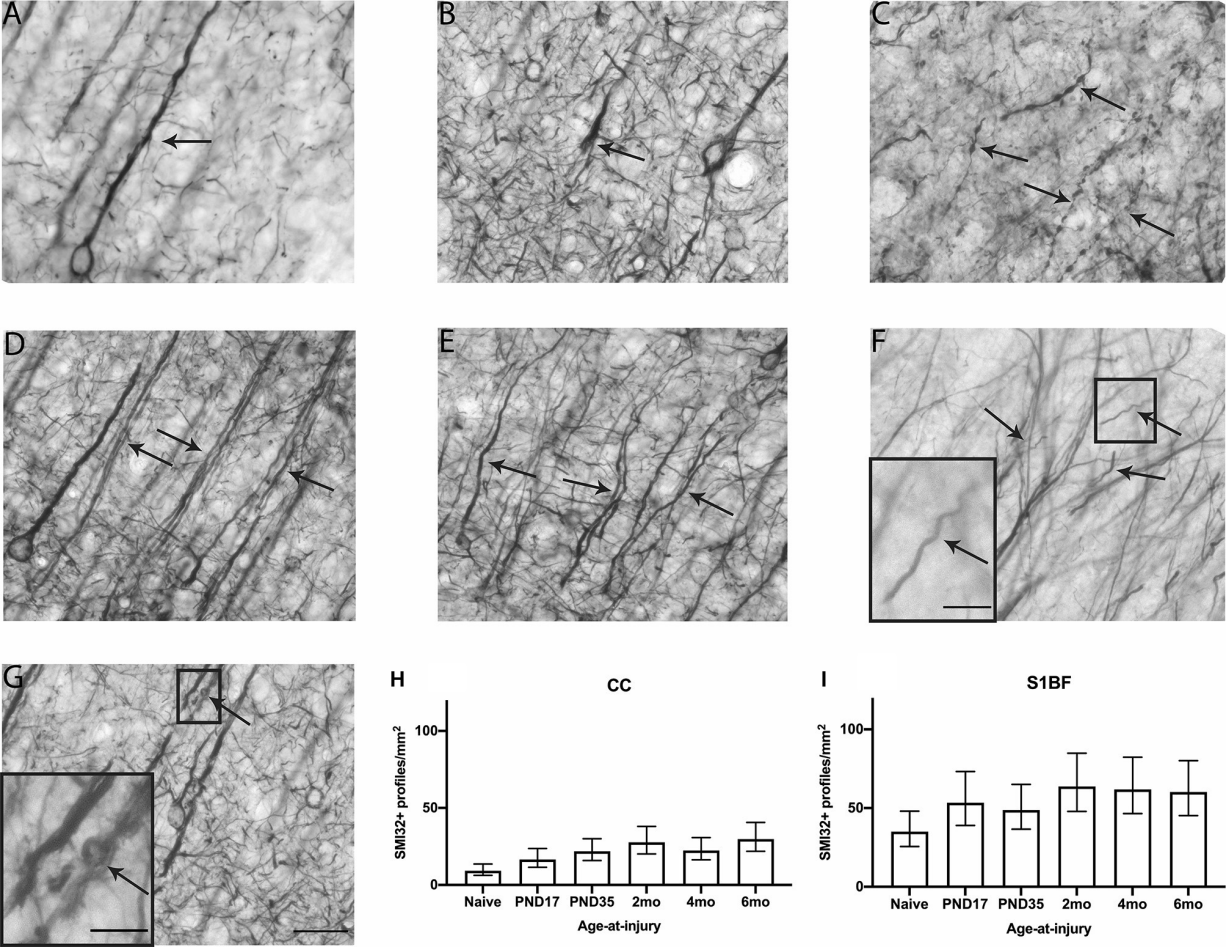
SMI32 dendritic profiles were quantified in the CC and S1BF. Indicative images of healthy dendrites **(A)** and dendritic pathology visualized by SMI32 immunohistochemistry were taken in the primary sensory barrel field (S1BF) of rats brain-injured at 6-months of age. Quantitative analysis included bulbs **(B)**, blebbing **(C)**, thickened axons (white arrows; **D**), or undulated axons (black arrows; **D**), thickened and undulated axons **(E)**, corkscrews **(F)**, and rings **(G)** indicated by the arrows (scale bar = 50 μm, inset = 10 μm). Dendritic pathology was expressed as the number of SMI32+ profiles/mm^2^ (mean ± 95%CI) in the corpus callosum (CC) and S1BF. Significantly more SMI32+ profiles were observed following mFPI compared to naïves in the CC **(H)**, regardless of whether the TBI was recent or remote. However, a greater extent of SMI32 pathology in the S1BF **(I)** compared with naïves was only evident with a more recent TBI (see Table 3).

To further examine cytoskeletal changes after TBI, immunohistochemistry against non-phosphorylated neurofilament heavy chain (SMI32) was used to identify dendritic neuronal pathology in the CC and S1BF. SMI32 immunoreactivity in healthy neurons of naïve brain appears as thin, straight fibers in dendrites with a slender outline around the perikarya in the S1BF (Figure 3A). Following brain-injury, SMI32 staining of pathology appeared as bulbs, blebbing, thickened, undulated as well as thickened and undulated dendrites, corkscrews, and rings in the S1BF (Figures 3B–G). Dendritic pathology was quantified in each image taken as the number of SMI32-positive (SMI32+) profiles per mm^2^ that occurred in both the CC and S1BF (Figures 3H,I). Animals in the injured groups had significantly more SMI32+ profiles than naïve ($\chi_{5}^{2}$ = 13.3; *p* < 0.05), but SMI32+ profiles in age-at-injury groups varied significantly by region ($\chi_{5}^{2}$ = 24.6; *p* < 0.001) (see Supplementary Table 3 for estimated counts and 95% CIs; Figures 3H,I). In the CC, brain-injured groups exhibited a significantly greater extent of SMI32+ profiles/mm^2^ compared with naïve (see Table 3). However, in the S1BF, only rats injured at 2-, 4-, and 6-months of age (recent brain injury) displayed a significantly greater extent of SMI32+ profiles/mm^2^ compared with naïve (see Table 3). These quantitative data implicate neurofilament dendritic pathology is apparent after a TBI, where the extent of pathology in gray matter of the S1BF is only greater than naïve controls with a more recent injury.

**Table 3:** SMI32 *post-hoc* contrasts between age-at-injury groups and naïve controls, separated by region, using the Dunnett's procedure.

**Contrast:** **age-at-injury (Dunnett)**	**Region**	**Co-efficient estimate**	**Lower 95% CI**	**Upper 95% CI**	***p*** **-values**
N vs. PND17	CC	0.5800	0.0483	1.112	**0.0326**
N vs. PND35	CC	0.8610	0.3574	1.364	**0.0009**
N vs. 2-months	CC	1.1000	0.5974	1.602	**<0.0001**
N vs. 4-months	CC	0.8890	0.3852	1.392	**0.0006**
N vs. 6-months	CC	1.1720	0.6737	1.670	**<0.0001**
N vs. PND17	S1BF	0.4200	−0.0249	0.865	0.0642
N vs. PND35	S1BF	0.3290	−0.0966	0.755	0.1293
N vs. 2-months	S1BF	0.5980	0.1725	1.023	**0.006**
N vs. 4-months	S1BF	0.5660	0.1419	0.991	**0.0091**
N vs. 6-months	S1BF	0.5400	0.1151	0.964	**0.0129**

*Bold means statistically significant p < 0.05*.

### The Extent of pTDP43 Neuronal Pathology Was Greater in Brain-Injured Groups Compared With Naïve in the S1BF and Hippocampus, Regardless of Whether the TBI Was Recent or Remote

**Figure 4:**
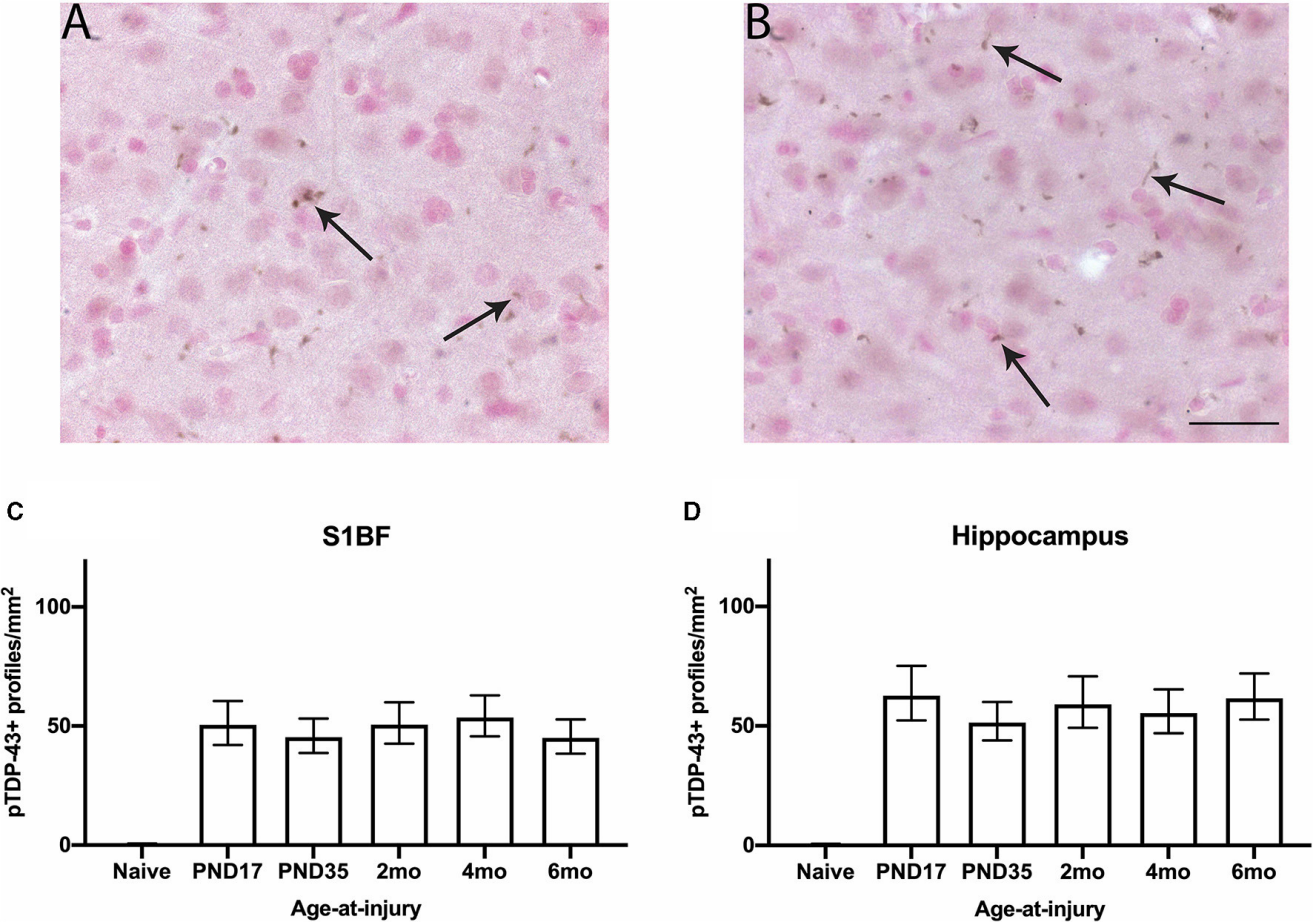
pTDP-43 neuronal profiles were quantified in the S1BF and hippocampus. Indicative images of neuronal pathology visualized by pTDP-43 immunohistochemistry were taken in the primary sensory barrel field (S1BF) of rats injured at 6-months of age. Quantitative analysis included nuclei inclusions **(A)** and cytoplasmic neurites **(B)** indicated by the black arrows (scale bar = 50 μm). Counts of neuronal pathology are expressed as the number of pTDP-43+ profiles/mm^2^ (mean ± 95%CI) in the S1BF and hippocampus. Significantly more pTDP-43+ profiles were observed following mFPI, regardless of age-at-injury, compared to naïves in both the S1BF **(C)** and the hippocampus **(D)**.

TAR DNA-binding protein 43 (TDP-43, transactive response DNA binding protein 43 kDa) primarily resides in the nucleus where it acts as a transcription factor and presents diffusely in the cytoplasm of the perikarya which is proposed to be involved with mRNA splicing and stability (57). The TDP-43 protein is known to become hyper-phosphorylated in pathological conditions, such as after a TBI, and aggregate within the nucleus of neurons, or translocate into the cytoplasm (58, 59). It is unclear how aggregated TDP-43 damages neurons other than reducing its functions in the nucleus (57). However, it is known that TDP-43 aggregation is detrimental to neurons as it is a hallmark of a number of neurodegenerative diseases including frontotemporal lobar dementia (FTLD) and amyotrophic lateral sclerosis (ALS) (60, 61). Immunohistochemistry against phosphorylated TDP-43 (pTDP-43) was conducted in order to visualize TDP-43 neuronal pathology in the S1BF and hippocampus. In the S1BF, pTDP-43+ profiles were identified as nuclei inclusions and cytoplasmic neurites (Figures 4A,B). Proteopathy of TDP-43 was quantified in each image as the number of phosphorylated TDP-43-positive (pTDP-43+) profiles per mm^2^ in the S1BF and hippocampus (Figures 4C,D). Brain-injured animals had significantly more pTDP-43+ profiles than naïve ($\chi_{5}^{2}$ = 149.2; *p* < 0.001) where pTDP-43+ profiles in age-at-injury groups did not significantly vary by region ($\chi_{5}^{2}$ = 7.6; *p* = 0.1788) (see Supplementary Table 4 for estimated counts and 95% CIs; see Figures 4C,D). The results indicate that TDP-43 is hyperphosphorylated in the S1BF and hippocampus, as detected in the nucleus and cytoplasm, after a single diffuse TBI regardless of whether the TBI was recent or remote. To examine whether age-at-injury influences further downstream aspects of the TBI molecular cascade, immunohistochemical analysis of glial activation was explored.

### No Differences in the Extent of GFAP Expressed by Astrocytes Was Evident Between Brain-Injured and Naïve Groups, Regardless of Whether the TBI Was Recent or Remote

**Figure 5:**
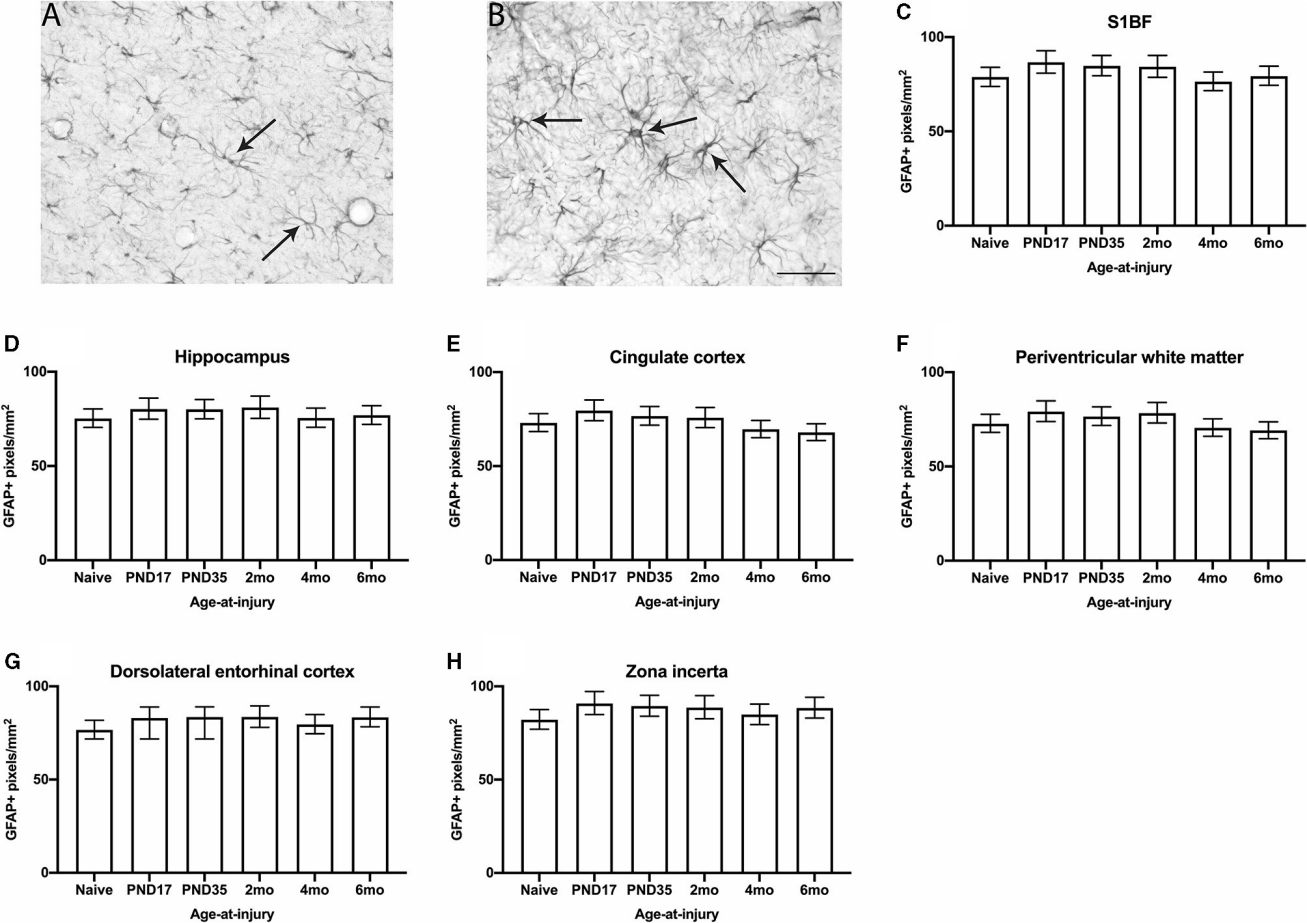
GFAP immunoreactivity was quantified in the S1BF, hippocampus, cingulate cortex, periventricular white matter, dorsolateral entorhinal cortex, and zona incerta. Representative images of ramified **(A)** and hypertrophic **(B)** astrocytes, indicated with black arrows (scale bar = 50 μm), visualized by GFAP immunohistochemistry were taken in the primary sensory barrel field (S1BF) of rats injured at 6-months of age. The expression of GFAP increases when astrocytes become hypertrophic and, thus, activated. Therefore, the extent of GFAP immunoreactivity is used as a measure of astrocyte activation and is expressed as the number of GFAP-positive pixels/mm^2^ (mean ± 95%CI) in the S1BF, hippocampus, cingulate cortex, lateral ventricle, dorsolateral entorhinal cortex, and zona incerta. There were no significant differences in the percentage of GFAP+ profiles observed following mFPI, regardless of age-at-injury, compared to naïves in the S1BF **(C)**, hippocampus **(D)**, cingulate cortex **(E)**, periventricular white matter **(F)**, dorsolateral entorhinal cortex **(G)**, or the zona incerta **(H)**.

Glial fibrillary acidic protein is a structural protein present in astrocytes. In the S1BF, astrocytes typically appear in the healthy brain as star-shaped cells with fine and highly ramified processes [Figure 5A; (62)]. After an insult, astrocytes become activated, which involves hypertrophy with an enlarged perikarya, reduced process complexity, and increased expression of GFAP [Figure 5B; (62)]. Thus, the extent of GFAP expressed by astrocytes is used as a measure of astrocyte activation following injury. The extent of GFAP expressed by astrocytes was quantified in each image taken of the S1BF, hippocampus, cingulate cortex, lateral ventricle, dorsolateral entorhinal cortex, and zona incerta as the number of GFAP+ pixels per mm^2^ (Figures 5C–H). Animals in the injured groups showed no significant differences in GFAP immunoreactivity compared with naïve ($\chi_{5}^{2}$ = 8.4; p = 0.1374). Glial fibrillary acidic protein immunoreactivity was different between brain regions analyzed, for example S1BF compared to hippocampus. However, there was no effect of injury on these differences ($\chi_{25}^{2}$ = 50.5; *p* < 0.01) (see Supplementary Table 5 for estimated counts and 95% CIs; Figures 5C–H). These quantitative data demonstrate that the expression of GFAP by astrocytes is comparable to naïve at 4–9.5 months after injury. The current study cannot determine whether temporal changes in GFAP occurred post-injury as reported by others (63).

### The Proportion of Deramified Microglia Was Greater in Brain-Injured Groups Compared With Naïve in the S1BF and Hippocampus, Regardless of Whether the TBI Was Recent or Remote

**Figure 6:**
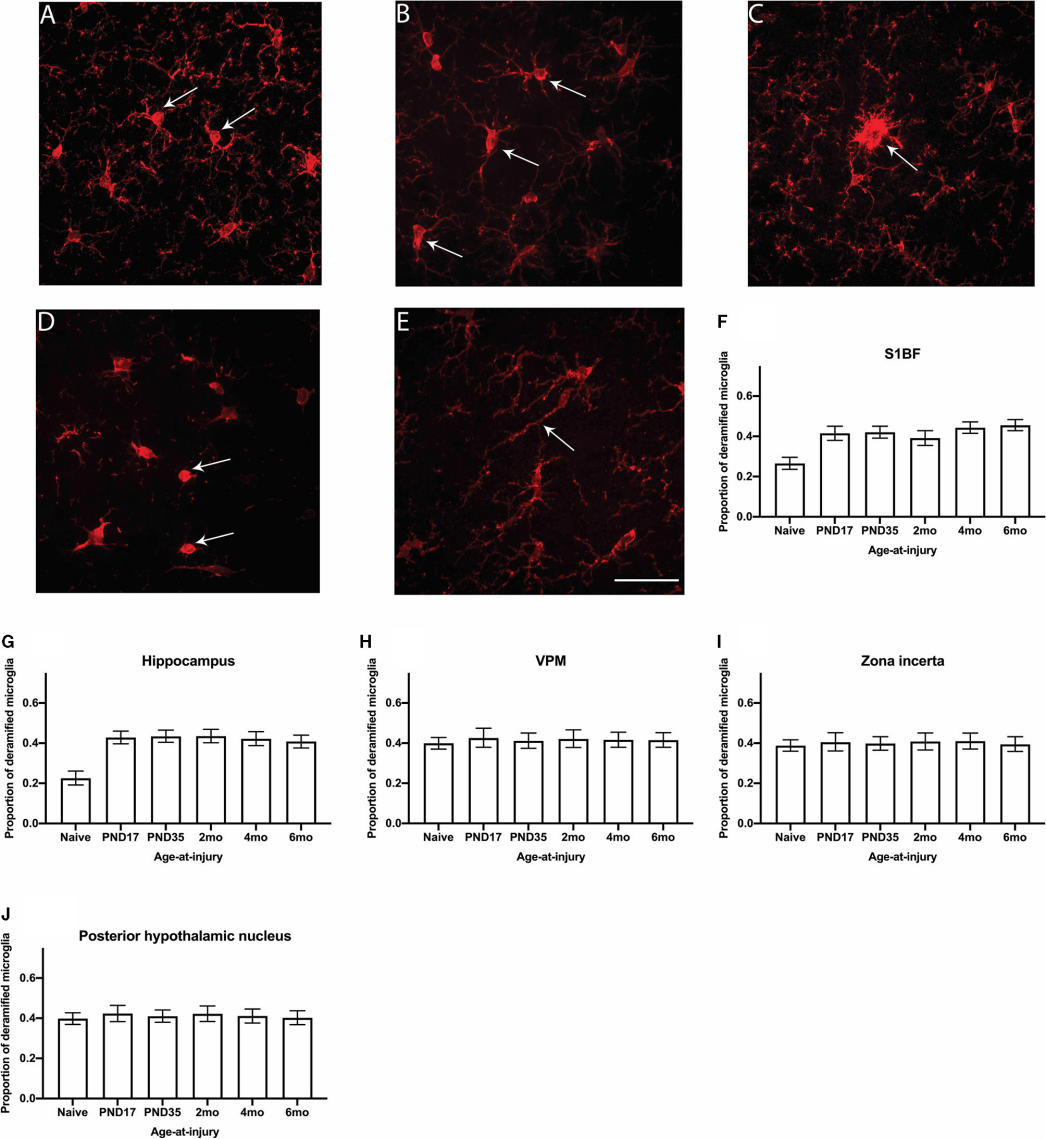
The proportion of deramified microglia were quantified in the S1BF, hippocampus, VPM, zona incerta, and posterior hypothalamic nucleus. Representative images of ramified **(A)** and deramified **(B–E)** microglia, indicated by the black arrows (scale bar = 50 μm), visualized by Iba1 immunohistochemistry were taken in the primary sensory barrel field (S1BF) of rats injured at 6-months of age. The proportion of deramified microglia (mean ± 95%CI) was determined in the S1BF, hippocampus, ventral posteromedial nucleus (VPM), zona incerta, and posterior hypothalamic nucleus. There was a significantly greater proportion of deramified microglia observed after brain injury, regardless of age-at-injury, compared to naïves in the S1BF **(F)**, hippocampus **(G)** but not in the zona incerta **(H)**, VPM **(I)**, or posterior hypothalamic nucleus **(J)**.

Ionized calcium binding adaptor molecule 1 (Iba1) is an actin-binding protein that is present in the microglial cytoskeleton and can be used to visualize microglia morphology (64). In the S1BF, microglia in the healthy brain typically appear in a ramified morphology with a small, round somata and long, thin, and highly branched processes [Figure 6A; (65)]. After an injury or infection, microglia undergo de-ramification where the cell body typically becomes swollen, and processes are retracted with reduced complexity. These morphologies are referred to as hyper-ramified, hypertrophic, and amoeboid [Figures 6B–D; (65)]. In addition to this, microglia have also been documented in aging and after an injury to have an elongated cell body with short processes along its length, referred to as rod microglia [Figure 6E; (66)]. Microglia morphologies were quantified in the S1BF, hippocampus, zone incerta, VPM, and posterior hypothalamic nucleus as the proportion of deramified microglia in relation to the total cell count in all four images per ROI per animal (Figures 6F–J). Animals in the injured groups had significantly greater proportion of deramified microglia than naïve ($\chi_{5}^{2}$ = 56.1; *p* < 0.001), but the proportion of deramified microglia in age-at-injury groups varied significantly by region ($\chi_{20}^{2}$ = 113.9; *p* < 0.001) (see Supplementary Table 6 for estimated counts and 95% CIs; Figures 6F–J). In the S1BF and hippocampus, brain-injured groups exhibited a significantly greater proportion of deramified microglia compared with naïve (see Table 4). However, in the VPM, zona incerta, and posterior hypothalamic nucleus, there were no significant differences in the proportion of deramified microglia between brain-injured groups compared to naïve (see Table 4). These quantitative data demonstrate that microglia become deramified after a single remote TBI, particularly in the S1BF and hippocampus.

**Table 4:** Deramified microglia *post-hoc* contrasts between age-at-injury groups and naïve controls, separated by region, using the Dunnett's procedure.

**Contrast:** **age-at-injury (Dunnett)**	**Region**	**Co-efficient estimate**	**Lower 95% CI**	**Upper 95% CI**	***p*** **-values**
N vs. PND17	S1BF	0.6760	0.4655	0.886	**<0.0001**
N vs. PND35	S1BF	0.6981	0.5027	0.894	**<0.0001**
N vs. 2-months	S1BF	0.5766	0.3605	0.793	**<0.0001**
N vs. 4-months	S1BF	0.7920	0.6001	0.984	**<0.0001**
N vs. 6-months	S1BF	0.8397	0.6502	1.029	**<0.0001**
N vs. PND17	Hippocampus	0.9485	0.7132	1.184	**<0.0001**
N vs. PND35	Hippocampus	0.9736	0.7407	1.207	**<0.0001**
N vs. 2-months	Hippocampus	0.9777	0.7382	1.217	**<0.0001**
N vs. 4-months	Hippocampus	0.9239	0.6814	1.166	**<0.0001**
N vs. 6-months	Hippocampus	0.8646	0.6257	1.103	**<0.0001**
N vs. PND17	VPM	0.1090	−0.1201	0.338	0.3511
N vs. PND35	VPM	0.0507	−0.1483	0.250	0.6175
N vs. 2-months	VPM	0.0926	−0.1247	0.310	0.4036
N vs. 4-months	VPM	0.0687	−0.1276	0.265	0.4927
N vs. 6-months	VPM	0.0665	−0.1266	0.259	0.4999
N vs. PND17	Zona incerta	0.0732	−0.1515	0.298	0.5232
N vs. PND35	Zona incerta	0.0436	−0.1419	0.229	0.6453
N vs. 2-months	Zona incerta	0.0833	−0.1307	0.297	0.4455
N vs. 4-months	Zona incerta	0.0913	−0.1122	0.295	0.3790
N vs. 6-months	Zona incerta	0.0276	−0.1674	0.223	0.7811
N vs. PND17	Posterior hypothalamic nucleus	0.1041	−0.0997	0.308	0.3167
N vs. PND35	Posterior hypothalamic nucleus	0.0515	−0.1216	0.225	0.5600
N vs. 2-months	Posterior hypothalamic nucleus	0.1010	−0.0959	0.298	0.3148
N vs. 4-months	Posterior hypothalamic nucleus	0.0537	−0.1329	0.240	0.5727
N vs. 6-months	Posterior hypothalamic nucleus	0.0192	−0.1670	0.205	0.8397

*Bold means statistically significant p < 0.05*.

### The Proportion of Microglia Colocalized With CD68 Was Greater for Brain-Injured Groups in the S1BF, VPM, and Zona Incerta, Regardless of Whether the TBI Was Recent or Remote

**Figure 7:**
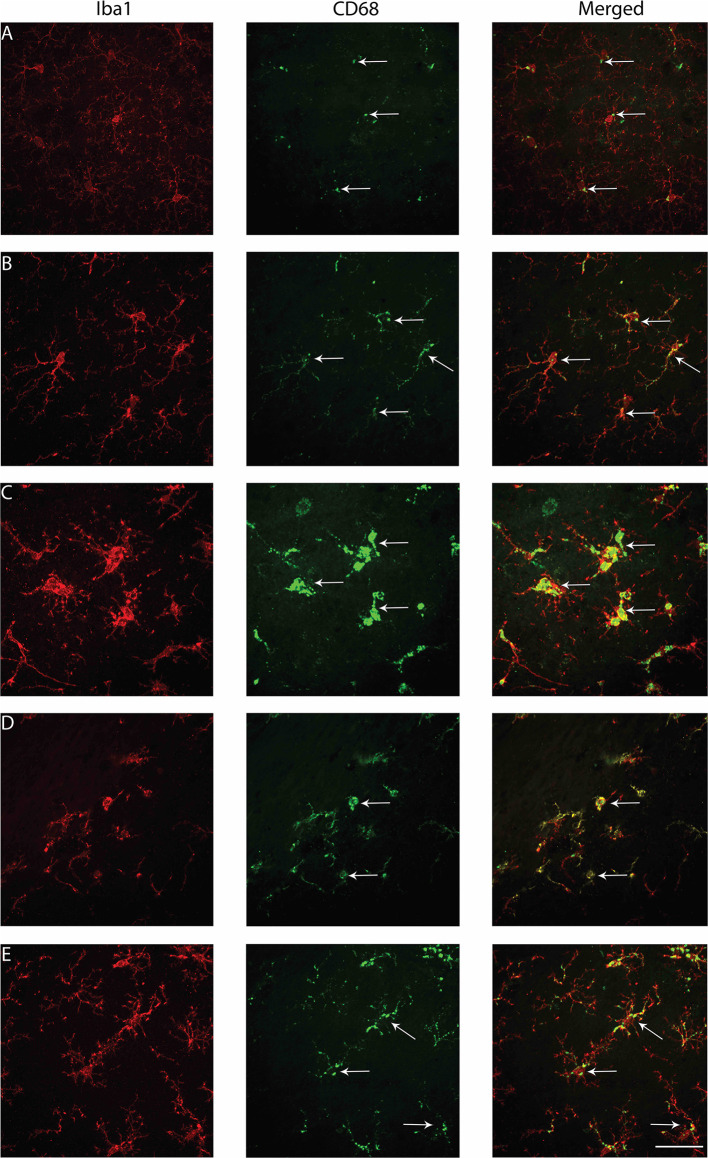
Examples of microglial morphologies colocalized with CD68 in the S1BF of rats brain-injured at 6-months of age. Representative images of microglial morphologies were taken in the primary sensory barrel field (S1BF) of rats injured at 6-months of age; ramified **(A)**, hyper-ramified **(B)**, hypertrophic **(C)**, amoeboid **(D)**, and rod **(E)**, and their colocalization with CD68 indicated with the white arrows (scale bar = 50 μm).

**Figure 8:**
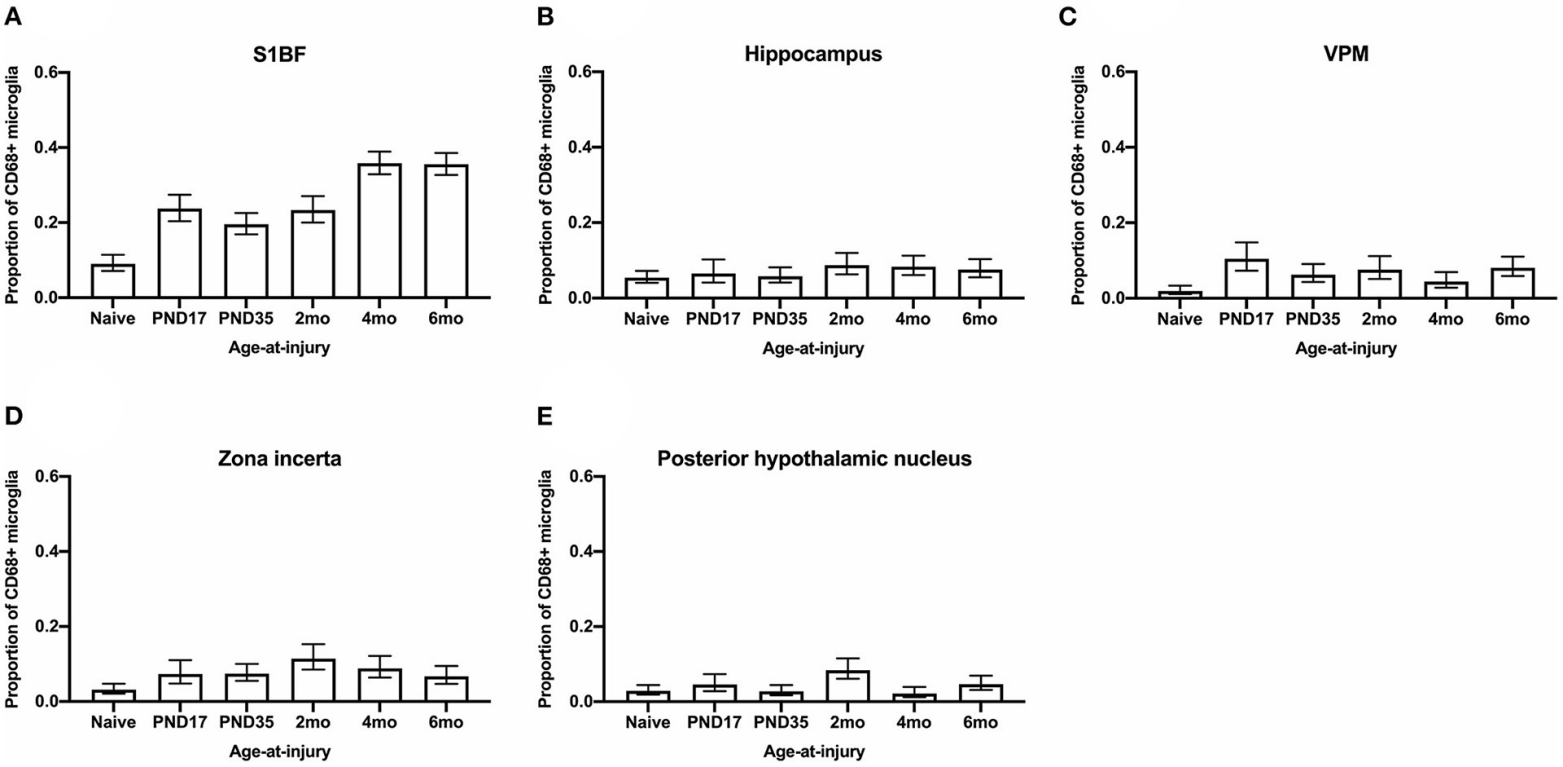
The proportion of microglia colocalized with CD68 were quantified in the S1BF, hippocampus, VPM, zona incerta, and posterior hypothalamic nucleus. Microglial colocalization with CD68 is expressed as the proportion of CD68+ microglia (mean ± 95%CI) in the primary sensory barrel field (S1BF), hippocampus, ventral posteromedial nucleus (VPM), zona incerta, and posterior hypothalamic nucleus. Significantly more CD68+ microglia were observed following mFPI compared to naïves in the S1BF **(A)**, zona incerta **(C)**, and VPM **(D)** regardless of whether the TBI was recent or remote. However, in the hippocampus **(B)** and posterior hypothalamic nucleus **(E)**, a greater proportion of microglia colocalized with CD68 was only evident compared with naïves with a more recent TBI (see Table 5).

Variations in the functional activation of microglia have been classified by the presence of specific immunohistochemical markers. To explore functional aspects of microglial activation, dual staining with ionizing actin-binding protein 1 (Iba1) and a surrogate marker of phagocytosis, cluster of differentiation 68 (CD68), was conducted. Microglia were counted in the S1BF, hippocampus, VPM, zona incerta, and posterior hypothalamic nucleus. In the S1BF, ramified microglia were evenly spread throughout each image taken per ROI and displayed minimal CD68 immunoreactivity (Figure 7A). Whilst pockets of hyper-ramified (Figure 7B) and deramified (Figure 7C) morphologies were observed and identified as CD68-positive (CD68+). Small numbers of amoeboid microglia were also present, and intermittently observed to colocalize with CD68 puncta (Figure 7D). Rod microglia, shown for the S1BF, aligned perpendicular to the dural surface (Figure 7E). Microglia colocalized with CD68 were quantified as the proportion of CD68+ microglia in relation to the total number of microglia counted in the four images taken of the S1BF, hippocampus, VPM, zona incerta, and posterior hypothalamic nucleus (Figures 8A–E). Brain-injured groups had significantly greater proportion of CD68+ microglia than naïve ($\chi_{5}^{2}$ = 67.4; *p* < 0.001), but the proportion of CD68+ microglia in age-at-injury groups varied significantly by region ($\chi_{20}^{2}$ = 106.1; *p* < 0.001) (see Supplementary Table 7 for estimated counts and 95% CIs; Figures 8A–E). In the S1BF, VPM, and zona incerta, brain-injured groups exhibited a significantly greater proportion of CD68+ microglia compared with naïve (see Table 5). However, in the hippocampus, only rats injured at 2- and 4-months of age had a significantly greater proportion of CD68+ microglia compared with naïve (see Table 5). In the posterior hypothalamic nucleus, the only significant difference was between rats brain-injured at 2-months of age compared with naïve (see Table 5). These results indicate that microglia colocalize with CD68 following a single diffuse brain injury, which differs in extent between age-at-injury groups in the hippocampus and posterior hypothalamic nucleus.

**Table 5:** CD68-positive microglia *post-hoc* contrasts between age-at-injury groups and naïve controls, separated by region, using the Dunnett's procedure.

**Contrast:** **age-at-injury (Dunnett)**	**Region**	**Co-efficient estimate**	**Lower 95% CI**	**Upper 95% CI**	***p*** **-Values**
N vs. PND17	S1BF	1.1468	0.8194	1.474	**<0.0001**
N vs. PND35	S1BF	0.9006	0.5808	1.220	**<0.0001**
N vs. 2-months	S1BF	1.1232	0.7917	1.455	**<0.0001**
N vs. 4-months	S1BF	1.7312	1.4358	2.027	**<0.0001**
N vs. 6-months	S1BF	1.7184	1.4241	2.013	**<0.0001**
N vs. PND17	Hippocampus	0.1959	−0.3804	0.772	0.5053
N vs. PND35	Hippocampus	0.0714	−0.4000	0.543	0.7666
N vs. 2-months	Hippocampus	0.5103	0.0476	0.973	**0.0306**
N vs. 4-months	Hippocampus	0.4599	0.0154	0.904	**0.0426**
N vs. 6-months	Hippocampus	0.3571	−0.0962	0.810	0.1226
N vs. PND17	VPM	1.7736	1.0977	2.450	**<0.0001**
N vs. PND35	VPM	1.2096	0.5259	1.893	**0.0005**
N vs. 2-months	VPM	1.4192	0.7234	2.115	**0.0001**
N vs. 4-months	VPM	0.8476	0.1219	1.573	**0.0221**
N vs. 6-months	VPM	1.4854	0.8377	2.133	**<0.0001**
N vs. PND17	Zona incerta	0.8935	0.2770	1.510	**0.0045**
N vs. PND35	Zona incerta	0.9005	0.3632	1.438	**0.001**
N vs. 2-months	Zona incerta	1.3784	0.8365	1.920	**<0.0001**
N vs. 4-months	Zona incerta	1.0947	0.5415	1.648	**0.0001**
N vs. 6-months	Zona incerta	0.7951	0.2296	1.361	**0.0059**
N vs. PND17	Posterior hypothalamic nucleus	0.4858	−0.1861	1.158	0.1564
N vs. PND35	Posterior hypothalamic nucleus	−0.0364	−0.6937	0.621	0.9135
N vs. 2-months	Posterior hypothalamic nucleus	1.1304	0.5619	1.699	**0.0001**
N vs. 4-months	Posterior hypothalamic nucleus	−0.3006	−1.0716	0.470	0.4447
N vs. 6-months	Posterior hypothalamic nucleus	0.5068	−0.1058	1.119	0.1049

*Bold means statistically significant p < 0.05*.

### The Proportion of Microglia Colocalized With TREM2 Was Greater in Brain-Injured Groups in the S1BF, Regardless of Whether the TBI Was Recent or Remote

**Figure 9:**
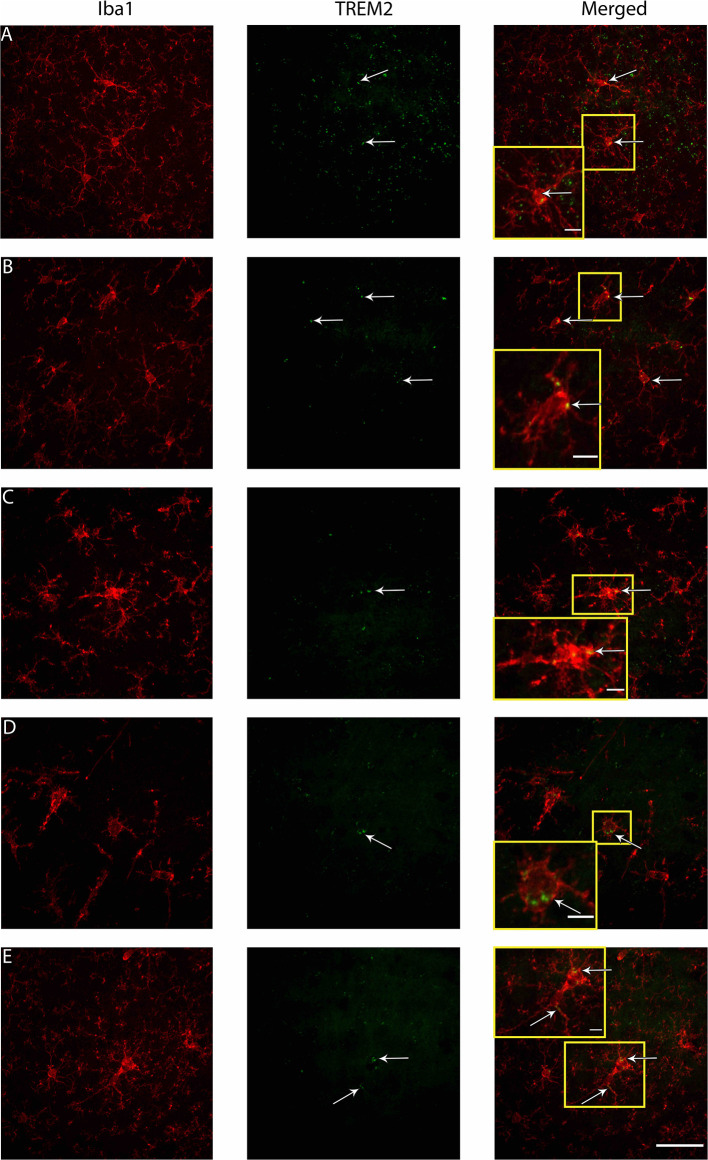
Examples of microglial morphologies in colocalization with TREM2 in the S1BF of rats brain-injured at 6-months of age. Representative images of microglial morphologies were taken in the primary sensory barrel field (S1BF) of rats injured at 6-months of age; ramified **(A)**, hyper-ramified **(B)**, deramified **(C)**, amoeboid **(D)**, and rod **(E)**, and their colocalization with TREM2 indicated with the white arrows (scale bar = 50 μm, inset = 10 μm).

**Figure 10:**
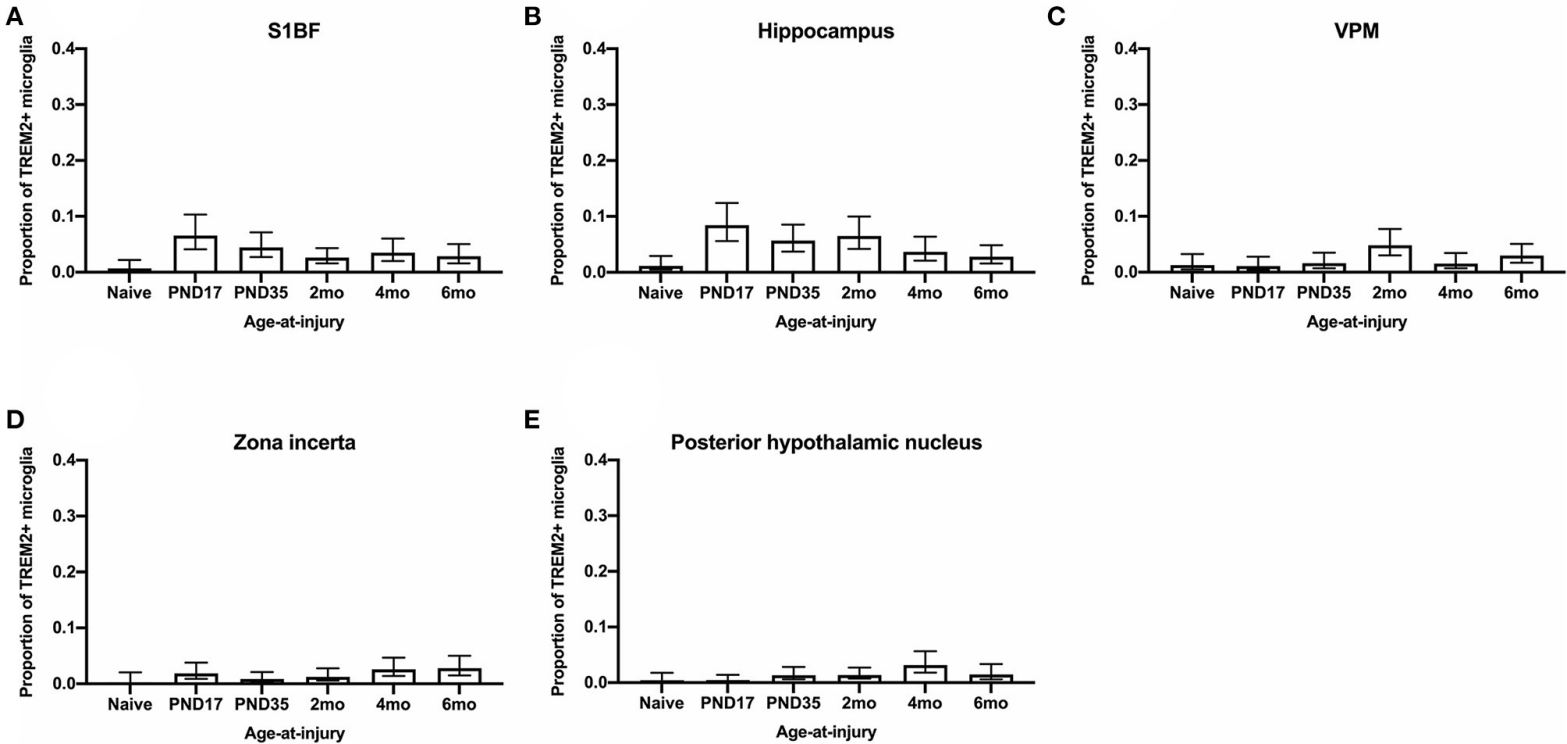
The proportion of microglia colocalized with TREM2 were quantified in the S1BF, hippocampus, VPM, zona incerta, and posterior hypothalamic nucleus. Microglial colocalization with TREM2 is expressed as the proportion of TREM2+ microglia (mean ± 95%CI) in the primary sensory barrel field (S1BF), hippocampus, ventral posteromedial nucleus (VPM), zona incerta, and posterior hypothalamic nucleus. Significantly more TREM2+ microglia were observed following mFPI compared to naïves in the S1BF **(A)**, regardless of whether the TBI was recent or remote. However, in the hippocampus **(B)**, a greater proportion of microglia colocalized with TREM2 was only evident compared with naïves with a more remote TBI (see Table 14). This trend is the opposite in the zona incerta **(C)**, VPM **(D)**, and posterior hypothalamic nucleus **(E)**, where a greater proportion of microglia colocalized with TREM2 was only evident compared with naïves with a more recent TBI (see Table 5).

Microglia express a range of cell surface receptors that detect specific particulate in the extracellular space. Triggering receptor expressed on myeloid cells 2 (TREM2) is a surface receptor that detects damage-associated lipid patterns associated with neurodegeneration (67, 68). Hence, dual immunohistochemical analysis of TREM2 was conducted with Iba1 to examine whether microglia may be utilizing this pathway to detect signals released from pathological neurons in regions where neuropathology is evident (S1BF, hippocampus) as well as connecting structures (zona incerta, VPM, posterior hypothalamic nucleus). In the S1BF, TREM2-positive (TREM2+) puncta were observed to intermittently colocalize with microglia (Figures 9A–E). The extent of TREM2 immunoreactivity by microglia was quantified as the proportion of TREM2+ microglia in relation to the total number of microglia counted in the four images taken in the center of either the S1BF, hippocampus, VPM, zona incerta, and posterior hypothalamic nucleus (Figures 10A–E). Brain-injured groups had significantly greater proportion of TREM2+ microglia than naïve ($\chi_{5}^{2}$ = 21.1; *p* < 0.001), but the proportion of TREM2+ microglia in age-at-injury groups varied significantly by region ($\chi_{20}^{2}$ = 69.7; *p* < 0.001) (see Supplementary Table 8 for estimated counts and 95% CIs; Figures 10A–E). In the S1BF, brain-injured groups exhibited a significantly greater proportion of TREM2+ microglia compared with naïve (see Table 6). However, in the hippocampus, only rats injured at PND17, PND35, 2-, and 4-months of age showed a significantly greater proportion of TREM2+ microglia compared with naïve animals. In the VPM, the only significant difference was between brain-injured groups at 2-months of age compared with naïve (see Table 6). Lastly, in the zona incerta and posterior hypothalamic nucleus, the only significant difference was between groups brain-injured at 4-months of age compared with naïve (see Table 6). Hence, these results suggest that microglia colocalize with TREM2 after both recent and remote TBI in the S1BF, but only after a recent brain injury in the VPM, zona incerta and posterior hypothalamic nucleus. Hence, neuroinflammation is determined by age-at-injury in specific brain regions.

**Table 6:** TREM2-positive microglia *post-hoc* contrasts between age-at-injury groups and naïve controls, separated by region, using the Dunnett's procedure.

**Contrast:** **age-at-injury (Dunnett)**	**Region**	**Co-efficient estimate**	**Lower 95% CI**	**Upper 95% CI**	***p*** **-values**
N vs. PND17	S1BF	2.3152	1.0349	3.600	**0.0004**
N vs. PND35	S1BF	1.9028	0.6177	3.190	**0.0037**
N vs. 2-months	S1BF	1.3532	0.0630	2.640	**0.0398**
N vs. 4-months	S1BF	1.6531	0.3400	2.970	**0.0136**
N vs. 6-months	S1BF	1.4503	0.1296	2.770	**0.0314**
N vs. PND17	Hippocampus	2.0622	1.0298	3.090	**0.0001**
N vs. PND35	Hippocampus	1.6377	0.6015	2.670	**0.0020**
N vs. 2-months	Hippocampus	1.7808	0.7321	2.830	**0.0009**
N vs. 4-months	Hippocampus	1.1813	0.0763	2.290	**0.0361**
N vs. 6-months	Hippocampus	0.8949	−0.2087	2.000	0.1120
N vs. PND17	VPM	−0.1559	−1.4825	1.170	0.8178
N vs. PND35	VPM	0.2218	−1.0166	1.460	0.7255
N vs. 2-months	VPM	1.3552	0.2834	2.430	**0.0132**
N vs. 4-months	VPM	0.2021	−1.0377	1.440	0.7493
N vs. 6-months	VPM	0.8558	−0.2397	1.950	0.1257
N vs. PND17	Zona incerta	1.8879	−0.2223	4.000	0.0795
N vs. PND35	Zona incerta	1.0958	−1.0898	3.280	0.3258
N vs. 2-months	Zona incerta	1.4893	−0.6475	3.630	0.1719
N vs. 4-months	Zona incerta	2.2212	0.1470	4.300	**0.0358**
N vs. 6-months	Zona incerta	2.3050	0.2331	4.380	0.0292
N vs. PND17	Posterior hypothalamic nucleus	0.0325	−1.7760	1.840	0.9719
N vs. PND35	Posterior hypothalamic nucleus	1.1355	−0.4536	2.720	0.1614
N vs. 2-months	Posterior hypothalamic nucleus	1.1290	−0.4308	2.690	0.1560
N vs. 4-months	Posterior hypothalamic nucleus	2.0028	0.4730	3.530	**0.0103**
N vs. 6-months	Posterior hypothalamic nucleus	1.2118	−0.4207	2.840	0.1457

*Bold means statistically significant p < 0.05*.

## Discussion

Previously it was accepted that those living with TBI exhibit fewer long-term symptoms if the brain injury event occurred at a juvenile age than in adulthood and later-life (69). In fact, older individuals are twice as likely to die from a brain injury than children (69). More recent research suggests that individuals who sustain a TBI in juvenile years will have different outcomes to those who sustain a TBI when older (16, 25, 70). For example, after a pediatric TBI, mice displayed social deficits in adulthood (71). And yet, the underlying neuropathology and glial activation across age-at-injury in relation to neurobehavioral function is only recently investigated (72, 73). Past research has focused on discrete time points post-injury rather than aging with injury. This research was conducted as part of a larger behavior study that subjected rats to diffuse brain injury at specific ages and evaluated behavioral performance at a fixed age (28). The current study aimed to determine whether the behavioral outcomes mapped to neuropathology and glial activation. To investigate neuropathological profiles, we evaluated APP, SMI34, SMI32, and glial changes using GFAP and Iba1 in conjunction with CD68 and TREM2 at 10-months of age across age-at-injury compared to naïve rats.

Overall, the results from this research demonstrate that there was a greater extent of neuropathology and proportion of microglial activation, but not astrogliosis, after a single diffuse TBI compared with naïve, which depended on brain region. Where the extent of neuropathology and proportion of microglial activation is similar in all brain-injured groups at 10-months of age regardless of whether the TBI was recent or remote. Amyloid precursor protein and SMI34 axonal pathology were investigated in the CC and S1BF where the extent of pathology was greater in all brain-injured groups when compared to naïve and did not differ with age-at-injury. pTDP-43 neuropathology was examined in the S1BF and hippocampus, where the extent of pathology was greater in all brain-injured groups, regardless of age-at-injury, compared with naïve. These findings indicate that a brain-injury in early-life may harbor detectable neuropathology in the gray and white matter that is equivalent to that observed with a TBI in adulthood. The proportion of deramified microglial morphologies, indicative of microglial activation, was also greater after a TBI, irrespective of age-at-injury, in the S1BF and hippocampus compared with naïve. However, this was not observed in the connecting structures: VPM, zona incerta, and posterior hypothalamic nucleus. Therefore, the proportion of microglial activation is greater in brain-injured groups compared with naïve in regions where neuropathology was also evident. As we did not examine neuropathology in the connecting regions it is unclear whether the lack of microglial activation in the VPM, zona incerta, and posterior hypothalamic nucleus was a result of neuropathological differences compared with the S1BF and hippocampus. Therefore, sustaining a TBI as a juvenile may hold a risk factor of neuropathology and microgliosis that could affect the onset or magnitude of neurological diseases later in life, such as dementia (74). A leading theory is that TBI results in neuropathology similar to aging that includes APP, neurofilament, and TDP-43 pathologies, as well as microglial changes, which promote premature disease onset (75).

The amount of dendritic neuropathology and microglial colocalization with functional markers depended on the whether the TBI was recent or remote and was region-specific. SMI32 dendritic pathology was investigated in the CC and S1BF where the extent of the pathology was greater in all brain-injured groups compared with naïve in the white matter of the CC, regardless of age-at-injury. However, there was only a greater extent of SMI32 pathology observed with more a recent brain-injury in the S1BF compared with naïve. These results demonstrate that the extent of neurofilament dendritic pathology is determined by age-at-injury in the cortical gray matter after a TBI. This is in contrast to the proportion of microglia colocalized with a surrogate marker of phagocytosis (CD68) and alternative activation (TREM2) in the S1BF, which was greater in all brain-injured groups compared with naïve, regardless of whether the TBI was recent or remote. Similar results were found for microglial colocalization with CD68 in the VPM and zona incerta. The proportion of microglia colocalized with CD68 and TREM2 was also determined by age-at-injury in a region-specific manner. Only the more recent TBI groups exhibited a greater proportion of CD68-positive microglia compared with naïve in the hippocampus and posterior hypothalamic nucleus. The groups with more remote brain-injury had a greater proportion of microglia colocalized with TREM2 in the hippocampus. However, this opposite trend was observed in the VPM, zona incerta and posterior hypothalamic nucleus, where the proportion of TREM2-positive microglia was only greater than naïve with a recent TBI. Therefore, in the hippocampus, microglia may exhibit a phagocytic role with a recent TBI and possibly shift to an alternatively activated phenotype as the TBI becomes more remote. While mFPI is not associated with overt cell death, we have evidence from our silver stain data that there is sub-acute neurodegeneration (33, 63). The golgi stain in our rats also supports that there is change in morphology of neurons that might require synaptic pruning (63). Microglia may also be phagocytizing neuronal debris in the aging brain as demonstrated in aged mice (76), thus, an increase in microglial phagocytosis may reflect a normal part of the aging process. It is unclear as to whether the differences in the microglial response between age-at-injury groups in connecting brain structures, VPM, zona incerta, and posterior hypothalamic nucleus, is a result of differences in neuropathology as this was not investigated in these regions. Differential responses of microglia phenotypes highlight the dynamic nature of microglial activation after a TBI at various stages of development.

Astrocytes are also involved with inflammatory cascades, alongside microglia, and form the glial scar around damaged tissue to barricade the injured area and promote tissue repair and resolution (77). Astrogliosis is typically reported to increase after a TBI in post-mortem tissue and experimental models (78), whilst in this study there were no significant differences in the extent of astrogliosis post-injury in any of the ROIs, quantified by the percentage of GFAP per mm^2^, compared to naïve or between the different ages at which the injury occurred. Studies in juvenile and young adult mice reported changes in astrocyte processes up to 30-days after a juvenile-closed head injury and moderate lateral FPI, respectively (79, 80). Specifically, after a TBI in juvenile mice, astrocyte processes became longer and thicker which initiated at the injury site and became evident in connecting brain regions with time post-injury (80). After a TBI in young adult mice, fewer astrocyte processes were directed toward the granular cell layer of the hippocampus than astrocytes of uninjured tissue. This study concluded that astrocytes alter their morphology which may lead to detrimental changes to astrocytic scaffolding of neurons. There was evidence of astrocyte hypertrophy in this study, however, this was not specific to injured animals and also occurred in naïve therefore, this may be an artifact of the aging process as preciously described (81, 82). A more detailed analysis of astrocyte morphology is required to determine whether changes in the morphology of astrocytes occurs with aging and after a TBI. As astrocyte activation did not coincide with microglial activation, we hypothesize that microglia can become activated independently of astrogliosis as similar results have been reported in different brain regions of mice 20-months after a CCI (83). However, we cannot determine if one precedes or follows the other using the current study design, this could only be achieved by including acute, sub-acute and chronic timepoints post-injury.

These immunohistochemical analyses were conducted as part of a previously published behavior study (28). Chronic functional outcomes of anxiety-like behavior and spatial memory were examined at 8-, 9-, and 10-months of age. There were no differences in ability for naïve and injured rats to walk around the open field area, however, injured rats went into the center of the arena fewer times than naïve. Brain-injured rats also spent less time in the center of the arena compared with naïve, where brain-injured groups at 2-, 4-, and 6-months spent the least amount of time in the center of the arena. Brain-injured rats, particularly those with a more recent injury, resided at the edges of the open field arena at 8-months of age which is representative of anxiety-like behavior (84). However, at 10-months of age there were no differences in anxiety-like behavior detected using the forced swim test, regardless of age-at-injury. Spatial memory was assessed at 8-, 9-, and 10- months of age via the novel object recognition test, although differences were observed at 8- and 9- months of age by 10-months rats explored the novel object for a similar time as naïve. These data suggest that by 10-months of age spatial memory deficits had resolved regardless of age-at-injury. However, the absence of functional deficits at 10-months of age does not directly align with the histopathological findings of the current study where neuropathology and microglial activation are evident with differences observed between age-at-injury groups. For example, spatial memory and anxiety are processed within the hippocampus (85) and yet a greater extent of pTDP-43 neuronal pathology and proportion of activated microglia was observed in brain-injured hippocampus, regardless of age-at-injury, at 10-months of age compared with naïve. The proportion of microglia colocalized with TREM2 was also greater in the hippocampus of brain-injured groups, particularly in those with a remote TBI, compared with naïve. Therefore, the observed pathological load may not reach a level of phenotypic expression regarding the ability to perform the novel object recognition and forced swim tests. Thus, more sensitive behavior tests may be required, as clinically observations indicate lasting and often subtle symptoms following a TBI that include depression as well as memory and cognitive deficits (86–88).

At acute and chronic time points, the glial response was associated exclusively with axonal degeneration in mice after a lateral FPI in adolescence (1.5-months), adulthood (3-months), and older age [12-months; (17)]. Furthermore, the axonal degeneration was worse with increasing age-at-injury, suggesting that the young brain can resolve neuropathology more effectively (17). The discrepancy in the published and present results may result from the use a more focal brain injury model which includes contusion and primary mechanical injury that is exacerbated with age-at-injury. The diffuse brain injury used in the present research involves predominantly secondary injury cascades which has been shown here to be more prominent, long-term, after a TBI in early-life compared to adulthood. The effect of a secondary injury in adulthood after experiencing an initial TBI as a juvenile has been examined in mice using the weight-drop injury model (89). Mice injured as a juvenile (PND35) exhibited improved pathological and functional outcomes after a second brain-injury in adulthood (PND70), compared with animals that received a single TBI at PND70 (89). The bone structure of the skull directly above the injury site was observed to have increased volume and resistance to torsion after a juvenile TBI that further increased with time post-injury compared with naïve mice (89). One interpretation of these results is that the strengthening of the skull after a juvenile brain-injury protects against further damage from a second injury in adulthood and, thus, improves pathological and functional outcomes (89). Therefore, a TBI in early-life may result in neuropathology and microglial activation, as shown in this study, which could increase the risk of developing age-related conditions. However, an initial brain-injury as a juvenile may protect against further damage from a secondary injury later in life as previously described (89), though, further research is required to confirm these results.

One of the major limitations of this study is the use of all male rats. This study utilized a single cohort of animals (*n* = 81) in order to assure identical exposure to housing and seasonal conditions. Therefore, all 81 male rats were processed at same time and if we also included females, it would become practically infeasible to process such a large number of animals. However, there is increasing research demonstrating that there are sex differences in behavioral and pathological outcomes after a TBI in the clinic and laboratory (90–94). Research has shown that males exhibit greater white matter damage than that observed in females after a TBI using diffusion-tensor imaging (90). In contrast to this, recent studies have demonstrated that females show more severe symptoms following TBI compared with males (94). Further studies are required to examine the effect of age-at-injury upon behavioral and pathological outcomes between sexes.

Another compromise of using a single cohort of animals in this study was that pathology was not examined at multiple time points post-injury, but only at 10-months. Since all animals were euthanized at the same age, this raises the question of whether changes to neuropathology and the glial response were dependent upon age-at-injury or time-since-injury. Due to the inability to distinguish between age-at-injury and time-since-injury effects in this study, it is unclear whether temporal pathogenesis changes with age-at-onset of injury. Looking at other studies, it has been extensively documented that neuropathology and glial activation is evident as early as 6-h following a TBI (95). Yet, findings are controversial with time post-injury, where neural and glial pathology has been reported years post-injury in some studies (4). Whilst neuropathology and gliosis have been observed to subside in the few weeks to months following brain injury compared with higher levels at acute time points (3, 96). Thus, these results indicate that there are peaks and troughs in the extent of neuropathology and glial activation with time following a TBI which we were not able to examine using the current study design. Future experiments could include the immunohistochemical markers used in this study to elucidate how age-at-injury influences acute, sub-acute, and chronic TBI-induced pathology.

We also need to explore neuropathology not just between brain-injured and naïve animals, but also with other models of disease. We know that neuronal and glial pathology observed after a TBI is also evident in neurodegenerative diseases such as Alzheimer's disease, ALS, CTE (97–99). For example, amyloid plaques, which are a hallmark pathological feature of AD, are also evident in a subset of individuals following a TBI which are suggested to be derived from the accumulation of APP in axons (100). Plaque deposition at 10-months of age in rat models of amyloid pathology has been demonstrated to result in loss of memory retention (101), which did was not evident in the current study. Therefore, the extent of neuropathology detected following a TBI in this study may not be enough to alter functional outcomes observed in disease. We also need to explore TBI-induced pathology in the context of aging. It has been previously demonstrated that a TBI in aged rats (24-months old) exhibit greater neuronal cell death than those injured as juveniles (102). In addition, others have reported higher mortality and greater neurological deficits at acute time points following TBI in 20-month-old rats compared with those injured in adulthood (69, 103). Therefore, in order to understand the burden of TBI-induced pathology observed in this study, it is necessary to compare the extent of pathology after a TBI to that observed with age and in disease models.

Other areas of neuropathology to investigate between sexes, with multiple time points post-injury compared with age and disease models include changes to cell number and volume of brain structures. Volumetric changes in the size of brain regions such as the hippocampus, cerebral white matter, and lateral ventricles have been identified at acute and sub-acute time points after a TBI in humans and in rodent focal injury models using magnetic resonance imaging (MRI) which are demonstrated to be a predictor of functional outcomes (104, 105). However, limited studies have investigated the volume of brain structures following diffuse brain injury in rats (106, 107) and at chronic time points following injury. Changes to cell number has also not yet been explored at chronic time points post-injury but acutely and sub-acutely we have data to support that there is an increase in the number of microglia and a decrease of neurons in the cortex of rodents using this injury model (33, 108). Unfortunately, the processes used to collect the tissue for the current paper prevented volumetric analysis and stereological cell counts. Additionally, a more in-depth glial analysis looking at morphological changes of astrocytes and microglia would be valuable in order to create a more complete picture of TBI pathophysiology with respect to the age at which injury occurred.

## Conclusion

In conclusion, the results from this study further the notion that age-at-injury plays a role in the extent of neuropathology after a TBI. Effective treatment strategies and prediction of long-term outcomes after a brain-injury will need to consider the age at which the injury occurred. This study examines aging with a brain injury via immunohistochemistry which was part of a broader study investigating the effects of age-at-injury on behavioral performance across time post-injury. Although no behavioral phenotype was evident in brain-injured rats at 10-months of age compared with naïve, there was a greater extent of neuropathology and proportion of activated microglia, regardless of whether the TBI was recent or remote. In addition to this, the extent of dendritic neurofilament pathology, and proportion of microglial colocalization with functional markers after a TBI was determined by age-at-injury in a region-specific manner. The distinct pattern of pathological features after a brain-injury at discrete stages of development may influence the risk of developing neurodegenerative diseases in later life. Additional research is recommended to distinguish between the effects of age-at-injury and time-since-injury as well as explore possible sex differences.

## Data Availability Statement

The original contributions presented in the study are included in the article/Supplementary Files, further inquiries can be directed to the corresponding author/s.

## Ethics Statement

The animal study was reviewed and approved by Institutional Animal Care and Use Committees (IACUC) at the University of Arizona (Tucson, AZ, USA).

## Author Contributions

PDA and JL conceived the study framework. RR and JZ performed the fluid percussion injuries and collected the brain tissue for this study. YD conducted the immunohistochemistry, imaging, and quantitative analysis. YD wrote an initial draft of the manuscript and formulated the figures and tables, with input and feedback from all authors. RR, JL, and JZ edited and revised the manuscript for final publication. All authors contributed to the article and approved the submitted version.

## Funding

Research reported in this manuscript was supported by the Arizona Alzheimer's Consortium, with matching funds from the University of Arizona, College of Medicine – Phoenix and Phoenix Children's Hospital. Partial support was provided by National Institute of Neurological Disorders and Stroke of the National Institutes of Health under award number R01 NS-065052, Fraternal Order of Eagles, and the J.O. and J.R. Wicking Trust. RR was supported by the Bisgrove Scholar award funded by Science Foundation Arizona. RR completed a portion of these studies as part of the National Neurotrauma Society WINTR-VISA.

## Conflict of Interest

The authors declare that the research was conducted in the absence of any commercial or financial relationships that could be construed as a potential conflict of interest.

## Publisher's Note

All claims expressed in this article are solely those of the authors and do not necessarily represent those of their affiliated organizations, or those of the publisher, the editors and the reviewers. Any product that may be evaluated in this article, or claim that may be made by its manufacturer, is not guaranteed or endorsed by the publisher.
