# Supplementary figures and images for: Age-at-Injury Determines the Extent of Long-Term Neuropathology and Microgliosis After a Diffuse Brain Injury in Male Rats
Source: Front Neurol. 2021 Sep 8;12:722526. doi: 10.3389/fneur.2021.722526 (PMC8455817; doi:10.3389/fneur.2021.722526)

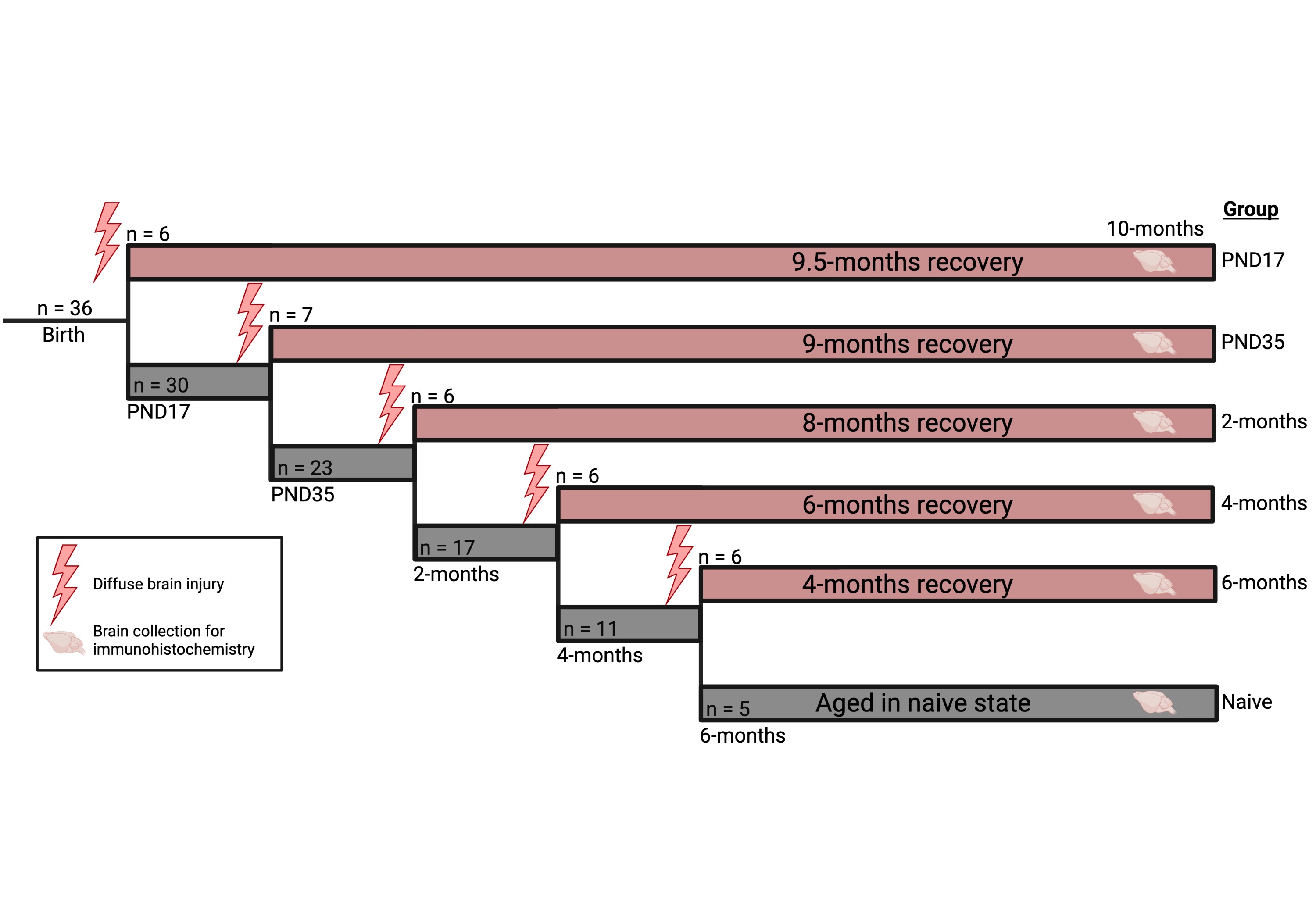

Supplement: Supplementary file 1 [file Image_1.JPEG]
