# Supplementary material for: Age-at-Injury Determines the Extent of Long-Term Neuropathology and Microgliosis After a Diffuse Brain Injury in Male Rats
Source: Front Neurol. 2021 Sep 8;12:722526. doi: 10.3389/fneur.2021.722526 (PMC8455817; doi:10.3389/fneur.2021.722526)
Supplement: Supplementary file 2 [file Image_2.pdf]

2 Supplementary Figures

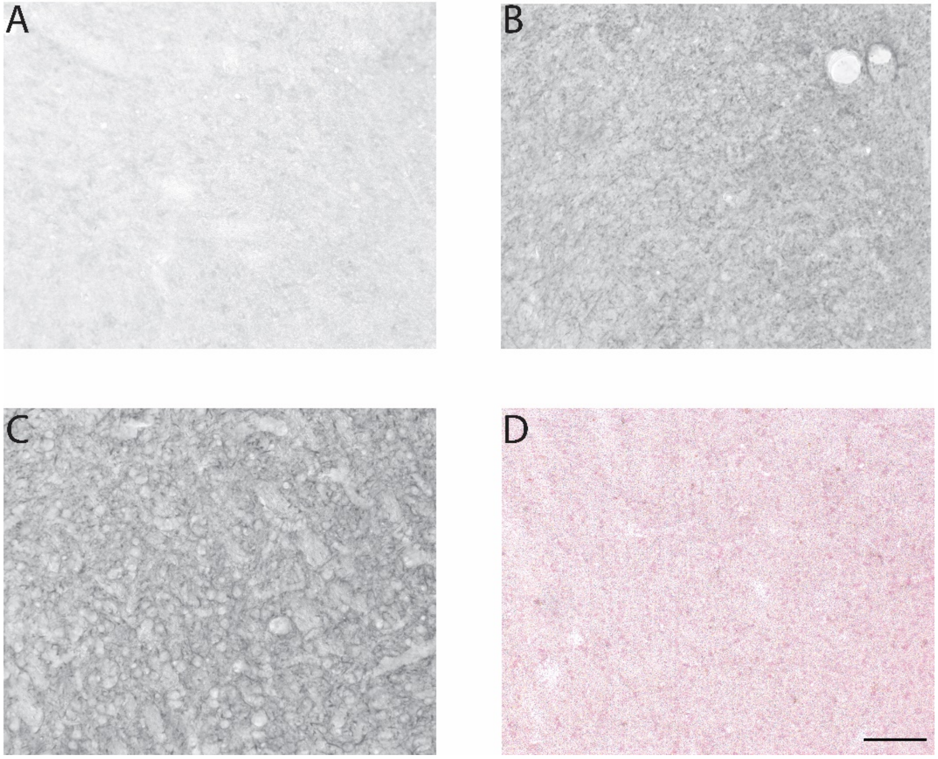

Supplementary Figure 1: ***Examples of APP, SMI34, SMI32 and pTDP-43 staining in the VPM of rats brain-injured at 6-months of age.*** *Representative images of APP (A), SMI34 (B), SMI32 (C) and pTDP-43 (D) staining taken in the ventral posteromedial (VPM) nucleus of rats injured at 6-months of age (scale bar = 100 m) where no overt pathology was observed.*
