# Supplementary material for: Age-at-Injury Determines the Extent of Long-Term Neuropathology and Microgliosis After a Diffuse Brain Injury in Male Rats
Source: Front Neurol. 2021 Sep 8;12:722526. doi: 10.3389/fneur.2021.722526 (PMC8455817; doi:10.3389/fneur.2021.722526)
Supplement: Supplementary file 3 [file Table_1.docx]

Supplementary Material

## Supplementary tables

**Supplementary Table 1:** *APP+ profiles/mm^2^ were counted in the corpus callosum (CC) and primary sensory barrel field (S1BF).*

| **Group** | **Region** | **Response** | **Lower 95% CI** | **Upper 95% CI** |
| --- | --- | --- | --- | --- |
| Naïve | CC | 7.3100 | 5.7600 | 9.280 |
| PND17 | CC | 49.8500 | 39.5100 | 62.900 |
| PND35 | CC | 51.7200 | 42.5800 | 62.810 |
| 2-months | CC | 49.0900 | 40.4400 | 59.600 |
| 4-months | CC | 59.2800 | 48.7700 | 72.060 |
| 6-months | CC | 48.9800 | 40.3500 | 59.460 |
| Naïve | S1BF | 11.5500 | 9.2100 | 14.490 |
| PND17 | S1BF | 78.8200 | 62.8000 | 98.910 |
| PND35 | S1BF | 81.7600 | 67.9000 | 98.450 |
| 2-months | S1BF | 77.6100 | 64.4300 | 93.490 |
| 4-months | S1BF | 93.7200 | 77.9500 | 112.690 |
| 6-months | S1BF | 77.4300 | 64.2100 | 93.380 |

**Supplementary Table 2:** *SMI34+ profiles/mm^2^ were counted in the corpus callosum (CC) and primary sensory barrel field (S1BF).*

| **Group** | **Region** | **Response** | **Lower 95% CI** | **Upper 95% CI** |
| --- | --- | --- | --- | --- |
| Naïve | CC | 17.2000 | 12.9000 | 22.800 |
| PND17 | CC | 44.0000 | 33.8000 | 57.300 |
| PND35 | CC | 35.5000 | 27.9000 | 45.200 |
| 2-months | CC | 44.6000 | 33.6000 | 59.100 |
| 4-months | CC | 36.1000 | 27.8000 | 46.700 |
| 6-months | CC | 43.1000 | 33.9000 | 54.900 |
| Naïve | S1BF | 46.8000 | 35.5000 | 61.500 |
| PND17 | S1BF | 119.8000 | 92.6000 | 155.000 |
| PND35 | S1BF | 96.7000 | 76.8000 | 121.800 |
| 2-months | S1BF | 121.4000 | 92.5000 | 159.300 |
| 4-months | S1BF | 98.2000 | 76.6000 | 126.000 |
| 6-months | S1BF | 117.4000 | 93.2000 | 147.800 |

**Supplementary Table 3:** *SMI32+ profiles/mm^2^ were counted in the corpus callosum (CC) and primary sensory barrel field (S1BF).*

| **Group** | **Region** | **Response** | **Lower 95% CI** | **Upper 95% CI** |
| --- | --- | --- | --- | --- |
| Naïve | CC | 12.6000 | 9.3300 | 17.000 |
| PND17 | CC | 20.1000 | 14.6600 | 27.500 |
| PND35 | CC | 20.4000 | 15.3100 | 27.200 |
| 2-months | CC | 26.1000 | 19.5200 | 34.800 |
| 4-months | CC | 24.3000 | 18.2300 | 32.500 |
| 6-months | CC | 25.7000 | 19.2500 | 34.300 |
| Naïve | S1BF | 31.3000 | 23.2600 | 42.100 |
| PND17 | S1BF | 49.9000 | 36.5600 | 68.100 |
| PND35 | S1BF | 50.7000 | 38.1700 | 67.500 |
| 2-months | S1BF | 64.8000 | 48.6900 | 86.200 |
| 4-months | S1BF | 60.4000 | 45.4800 | 80.300 |
| 6-months | S1BF | 63.8000 | 48.0100 | 84.800 |

**Supplementary Table 4:** *pTDP-43+ profiles/mm^2^ were counted in the primary sensory barrel field (S1BF) and hippocampus.*

| **Group** | **Region** | **Response** | **Lower 95% CI** | **Upper 95% CI** |
| --- | --- | --- | --- | --- |
| Naïve | S1BF | 0.9110 | 0.6280 | 1.320 |
| PND17 | S1BF | 52.7090 | 44.8330 | 61.970 |
| PND35 | S1BF | 45.3520 | 39.4320 | 52.160 |
| 2-months | S1BF | 51.2370 | 43.9450 | 59.740 |
| 4-months | S1BF | 51.3290 | 44.5080 | 59.200 |
| 6-months | S1BF | 49.5290 | 43.0270 | 57.010 |
| Naïve | Hippocampus | 1.0760 | 0.7410 | 1.560 |
| PND17 | Hippocampus | 62.2530 | 52.9550 | 73.180 |
| PND35 | Hippocampus | 53.5640 | 46.5930 | 61.580 |
| 2-months | Hippocampus | 60.5150 | 51.7890 | 70.710 |
| 4-months | Hippocampus | 60.6240 | 52.4750 | 70.040 |
| 6-months | Hippocampus | 58.4970 | 50.8630 | 67.280 |

**Supplementary Table 5:** *GFAP+ pixels were counted in the primary sensory barrel field (S1BF) and hippocampus, cingulate cortex, periventricular white matter, dorsolateral entorhinal cortex and zona incerta.*

| **Group** | **Region** | **Response** | **Lower 95% CI** | **Upper 95% CI** |
| --- | --- | --- | --- | --- |
| Naïve | S1BF | 78.5000 | 74.1000 | 83.100 |
| PND17 | S1BF | 85.5000 | 80.4000 | 90.900 |
| PND35 | S1BF | 84.1000 | 79.4000 | 89.000 |
| 2-months | S1BF | 84.2000 | 79.1000 | 89.500 |
| 4-months | S1BF | 78.1000 | 73.7000 | 82.700 |
| 6-months | S1BF | 79.6000 | 75.2000 | 84.300 |
| Naïve | Hippocampus | 75.1000 | 70.9000 | 79.600 |
| PND17 | Hippocampus | 81.8000 | 76.9000 | 87.000 |
| PND35 | Hippocampus | 80.4000 | 75.9000 | 85.200 |
| 2-months | Hippocampus | 80.5000 | 75.7000 | 85.700 |
| 4-months | Hippocampus | 74.7000 | 70.5000 | 79.200 |
| 6-months | Hippocampus | 76.2000 | 71.9000 | 80.700 |
| Naïve | Cingulate cortex | 70.8000 | 66.8000 | 75.000 |
| PND17 | Cingulate cortex | 77.1000 | 72.4000 | 82.000 |
| PND35 | Cingulate cortex | 75.8000 | 71.5000 | 80.300 |
| 2-months | Cingulate cortex | 75.9000 | 71.3000 | 80.700 |
| 4-months | Cingulate cortex | 70.4000 | 66.5000 | 74.600 |
| 6-months | Cingulate cortex | 71.8000 | 67.8000 | 76.000 |
| Naïve | Periventricular white matter | 71.4000 | 67.4000 | 75.600 |
| PND17 | Periventricular white matter | 77.7000 | 73.1000 | 82.700 |
| PND35 | Periventricular white matter | 76.4000 | 72.2000 | 81.000 |
| 2-months | Periventricular white matter | 76.5000 | 71.9000 | 81.400 |
| 4-months | Periventricular white matter | 71.0000 | 67.1000 | 75.300 |
| 6-months | Periventricular white matter | 72.4000 | 68.4000 | 76.700 |
| Naïve | Dorsolateral entorhinal cortex | 78.5000 | 74.1000 | 83.200 |
| PND17 | Dorsolateral entorhinal cortex | 85.5000 | 80.4000 | 91.000 |
| PND35 | Dorsolateral entorhinal cortex | 84.1000 | 79.4000 | 89.100 |
| 2-months | Dorsolateral entorhinal cortex | 84.2000 | 79.2000 | 89.600 |
| 4-months | Dorsolateral entorhinal cortex | 78.2000 | 73.8000 | 82.800 |
| 6-months | Dorsolateral entorhinal cortex | 79.7000 | 75.2000 | 84.400 |
| Naïve | Zona incerta | 84.0000 | 79.3000 | 89.000 |
| PND17 | Zona incerta | 91.5000 | 86.1000 | 97.300 |
| PND35 | Zona incerta | 90.0000 | 85.0000 | 95.300 |
| 2-months | Zona incerta | 90.1000 | 84.7000 | 95.900 |
| 4-months | Zona incerta | 83.6000 | 79.0000 | 88.600 |
| 6-months | Zona incerta | 85.2000 | 80.5000 | 90.300 |

**Supplementary Table 6:** *Deramified microglia were counted in the primary sensory barrel field (S1BF) and hippocampus, ventral posteromedial (VPM) nucleus, zona incerta and posterior hypothalamic nucleus.*

| **Group** | **Region** | **Probability** | **Lower 95% CI** | **Upper 95% CI** |
| --- | --- | --- | --- | --- |
| Naïve | S1BF | 0.3460 | 0.3280 | 0.364 |
| PND17 | S1BF | 0.4180 | 0.3970 | 0.438 |
| PND35 | S1BF | 0.4120 | 0.3940 | 0.430 |
| 2-months | S1BF | 0.4130 | 0.3920 | 0.434 |
| 4-months | S1BF | 0.4190 | 0.4010 | 0.438 |
| 6-months | S1BF | 0.4160 | 0.3980 | 0.434 |
| Naïve | Hippocampus | 0.3420 | 0.3240 | 0.361 |
| PND17 | Hippocampus | 0.4130 | 0.3930 | 0.434 |
| PND35 | Hippocampus | 0.4080 | 0.3900 | 0.427 |
| 2-months | Hippocampus | 0.4090 | 0.3880 | 0.429 |
| 4-months | Hippocampus | 0.4150 | 0.3950 | 0.435 |
| 6-months | Hippocampus | 0.4120 | 0.3930 | 0.431 |
| Naïve | VPM | 0.3610 | 0.3420 | 0.379 |
| PND17 | VPM | 0.4330 | 0.4110 | 0.456 |
| PND35 | VPM | 0.4280 | 0.4080 | 0.449 |
| 2-months | VPM | 0.4290 | 0.4060 | 0.451 |
| 4-months | VPM | 0.4350 | 0.4140 | 0.456 |
| 6-months | VPM | 0.4320 | 0.4110 | 0.452 |
| Naïve | Zona incerta | 0.3480 | 0.3300 | 0.366 |
| PND17 | Zona incerta | 0.4190 | 0.3970 | 0.442 |
| PND35 | Zona incerta | 0.4140 | 0.3940 | 0.434 |
| 2-months | Zona incerta | 0.4150 | 0.3930 | 0.437 |
| 4-months | Zona incerta | 0.4210 | 0.4000 | 0.442 |
| 6-months | Zona incerta | 0.4170 | 0.3970 | 0.438 |
| Naïve | Posterior hypothalamic nucleus | 0.3550 | 0.3380 | 0.373 |
| PND17 | Posterior hypothalamic nucleus | 0.4270 | 0.4060 | 0.449 |
| PND35 | Posterior hypothalamic nucleus | 0.4220 | 0.4030 | 0.441 |
| 2-months | Posterior hypothalamic nucleus | 0.4230 | 0.4020 | 0.444 |
| 4-months | Posterior hypothalamic nucleus | 0.4290 | 0.4090 | 0.449 |
| 6-months | Posterior hypothalamic nucleus | 0.4260 | 0.4060 | 0.445 |

**Supplementary Table 7:** *CD68-positive microglia were counted in the primary sensory barrel field (S1BF) and hippocampus, ventral posteromedial (VPM) nucleus, zona incerta and posterior hypothalamic nucleus.*

| **Group** | **Region** | **Probability** | **Lower 95% CI** | **Upper 95% CI** |
| --- | --- | --- | --- | --- |
| Naïve | Hippocampus | 0.0316 | 0.0259 | 0.039 |
| PND17 | Hippocampus | 0.0739 | 0.0612 | 0.089 |
| PND35 | Hippocampus | 0.0581 | 0.0487 | 0.069 |
| 2-months | Hippocampus | 0.0842 | 0.0707 | 0.100 |
| 4-months | Hippocampus | 0.0977 | 0.0834 | 0.114 |
| 6-months | Hippocampus | 0.1003 | 0.0860 | 0.117 |
| Naïve | Posterior hypothalamic nucleus | 0.0175 | 0.0138 | 0.022 |
| PND17 | Posterior hypothalamic nucleus | 0.0417 | 0.0334 | 0.052 |
| PND35 | Posterior hypothalamic nucleus | 0.0325 | 0.0263 | 0.040 |
| 2-months | Posterior hypothalamic nucleus | 0.0477 | 0.0386 | 0.059 |
| 4-months | Posterior hypothalamic nucleus | 0.0556 | 0.0457 | 0.068 |
| 6-months | Posterior hypothalamic nucleus | 0.0573 | 0.0472 | 0.069 |
| Naïve | S1BF | 0.1236 | 0.1064 | 0.143 |
| PND17 | S1BF | 0.2563 | 0.2283 | 0.287 |
| PND35 | S1BF | 0.2105 | 0.1883 | 0.235 |
| 2-months | S1BF | 0.2842 | 0.2557 | 0.315 |
| 4-months | S1BF | 0.3185 | 0.2945 | 0.343 |
| 6-months | S1BF | 0.3251 | 0.3015 | 0.350 |
| Naïve | VPM | 0.0257 | 0.0207 | 0.032 |
| PND17 | VPM | 0.0607 | 0.0493 | 0.074 |
| PND35 | VPM | 0.0476 | 0.0389 | 0.058 |
| 2-months | VPM | 0.0692 | 0.0568 | 0.084 |
| 4-months | VPM | 0.0805 | 0.0672 | 0.096 |
| 6-months | VPM | 0.0828 | 0.0693 | 0.099 |
| Naïve | Zona incerta | 0.0317 | 0.0258 | 0.039 |
| PND17 | Zona incerta | 0.0740 | 0.0611 | 0.089 |
| PND35 | Zona incerta | 0.0582 | 0.0485 | 0.070 |
| 2-months | Zona incerta | 0.0843 | 0.0704 | 0.101 |
| 4-months | Zona incerta | 0.0978 | 0.0831 | 0.115 |
| 6-months | Zona incerta | 0.1005 | 0.0857 | 0.117 |

**Supplementary Table 8:** *TREM2-positive microglia were counted in the primary sensory barrel field (S1BF) and hippocampus, ventral posteromedial (VPM) nucleus, zona incerta and posterior hypothalamic nucleus.*

| **Group** | **Region** | **Probability** | **Lower 95% CI** | **Upper 95% CI** |
| --- | --- | --- | --- | --- |
| Naïve | Hippocampus | 0.0143 | 0.0079 | 0.026 |
| PND17 | Hippocampus | 0.0601 | 0.0420 | 0.085 |
| PND35 | Hippocampus | 0.0481 | 0.0331 | 0.069 |
| 2-months | Hippocampus | 0.0570 | 0.0400 | 0.081 |
| 4-months | Hippocampus | 0.0562 | 0.0381 | 0.082 |
| 6-months | Hippocampus | 0.0482 | 0.0330 | 0.070 |
| Naïve | Posterior hypothalamic nucleus | 0.0037 | 0.0019 | 0.007 |
| PND17 | Posterior hypothalamic nucleus | 0.0160 | 0.0102 | 0.025 |
| PND35 | Posterior hypothalamic nucleus | 0.0127 | 0.0079 | 0.020 |
| 2-months | Posterior hypothalamic nucleus | 0.0151 | 0.0098 | 0.023 |
| 4-months | Posterior hypothalamic nucleus | 0.0149 | 0.0093 | 0.024 |
| 6-months | Posterior hypothalamic nucleus | 0.0127 | 0.0079 | 0.020 |
| Naïve | S1BF | 0.0097 | 0.0053 | 0.018 |
| PND17 | S1BF | 0.0414 | 0.0282 | 0.060 |
| PND35 | S1BF | 0.0330 | 0.0221 | 0.049 |
| 2-months | S1BF | 0.0392 | 0.0272 | 0.056 |
| 4-months | S1BF | 0.0387 | 0.0258 | 0.058 |
| 6-months | S1BF | 0.0330 | 0.0222 | 0.049 |
| Naïve | VPM | 0.0068 | 0.0037 | 0.013 |
| PND17 | VPM | 0.0294 | 0.0195 | 0.044 |
| PND35 | VPM | 0.0234 | 0.0152 | 0.036 |
| 2-months | VPM | 0.0279 | 0.0186 | 0.041 |
| 4-months | VPM | 0.0275 | 0.0178 | 0.042 |
| 6-months | VPM | 0.0234 | 0.0154 | 0.015 |
| Naïve | Zona incerta | 0.0046 | 0.0024 | 0.009 |
| PND17 | Zona incerta | 0.0201 | 0.0129 | 0.031 |
| PND35 | Zona incerta | 0.0159 | 0.0101 | 0.025 |
| 2-months | Zona incerta | 0.0190 | 0.0123 | 0.029 |
| 4-months | Zona incerta | 0.0187 | 0.0119 | 0.029 |
| 6-months | Zona incerta | 0.0160 | 0.0102 | 0.025 |
